# Tipping points induced by parameter drift in an excitable ocean model

**DOI:** 10.1038/s41598-021-90138-1

**Published:** 2021-05-27

**Authors:** Stefano Pierini, Michael Ghil

**Affiliations:** 1 Department of Science and Technology, Parthenope University of Naples, Centro Direzionale, Isola C4, 80143 Napoli, Italy; 2 CoNISMa, Rome, Italy; 3 Geosciences Department and Laboratoire de Météorologie Dynamique (CNRS and IPSL), École Normale Supérieure and PSL University, Paris, France; 4 Atmospheric and Oceanic Sciences Department, University of California at Los Angeles, Los Angeles, CA USA

**Keywords:** Climate sciences, Climate change

## Abstract

Numerous systems in the climate sciences and elsewhere are excitable, exhibiting coexistence of and transitions between a basic and an excited state. We examine the role of tipping between two such states in an excitable low-order ocean model. Ensemble simulations are used to obtain the model’s pullback attractor (PBA) and its properties, as a function of a forcing parameter $\gamma$ and of the steepness $\delta$ of a climatological drift in the forcing. The tipping time $t_{\mathrm{{tp}}}$ is defined as the time at which the transition to relaxation oscillations (ROs) arises: at constant forcing this occurs at $\gamma =\gamma _{\mathrm{c}}$. As the steepness $\delta$ decreases, $t_{\mathrm{{tp}}}$ is delayed and the corresponding forcing amplitude decreases, while remaining always above $\gamma _{\mathrm{c}}$. With periodic perturbations, that amplitude depends solely on $\delta$ over a significant range of parameters: this provides an example of rate-induced tipping in an excitable system. Nonlinear resonance occurs for periods comparable to the RO time scale. Coexisting PBAs and total independence from initial states are found for subsets of parameter space. In the broader context of climate dynamics, the parameter drift herein stands for the role of anthropogenic forcing.

## Introduction

Sudden changes in behavior of a physical system have played an important role in the geosciences since the early 1950s^1–4^; see also Ghil et al.^5^. The study of such changes was systematically described, under the name of bifurcations and associated regime changes^6,7^. More recently, the term “tipping points” has been introduced from the social sciences^8^ into the climate sciences by Lenton et al.^9^ and has attracted considerable attention.

A useful distinction between bifurcations and tipping points (TPs), beyond the rhetorical effect of the latter, does arise in the theory of dynamical systems with explicit time dependence in the forcing or coefficients. Such systems are opposed to those without explicit time dependence, that can often be treated within the framework of classical differentiable dynamical systems^10,11^. The latter are referred to as autonomous, the former in general as nonautonomous. For nonautonomous systems one often makes also the distinction between random forcing, which leads to random dynamical systems^12,13^, and purely deterministic forcing, which leads to dynamical systems that are also termed simply as nonautonomous^14^. A more general theory that combines the deterministic and random cases is also emerging^15^; Ghil & Lucarini^16^ provide a discussion of the latter in the climate context.

Kuehn^17^, among others, has shown how TPs generalize bifurcations in the broader setting of nonautonomous and random dynamical systems, by considering the detailed evolution of the system in the neighborhood of a TP, while Ashwin et al.^18^ have classified this behavior into three broad classes:

1. B-tipping or Bifurcation-due tipping—slow change in a parameter leads to the system’s passage through a classical bifurcation;
2. N-tipping or Noise-induced tipping—random fluctuations lead to the system’s crossing an attractor basin boundary; and
3. R-tipping or Rate-induced tipping—rapid changes lead to the system’s losing track of a slow change in its attractors.

Further perspective is provided by Feudel et al.^19^ and Ghil^20^ on climate applications and by Ashwin, Feudel, Wieczorek and coauthors^21–24^.

The autonomous differentiable dynamical system’s framework has served the climate sciences well for several decades^1–4,6,25^. It is mainly the recent interest in anthropogenic climate change and its interaction with the climate system’s natural variability that have led the research groups of Ghil and of Tél^26–30^, followed rapidly by others, to introduce the framework of nonautonomous and random dynamical systems into the climate sciences.

In the context of nonautonomous dynamical systems, we analyze in the present study a problem that has received relatively little attention to date: the tipping induced by a parameter drift in an excitable system, marking the transition from a stable basic state to self-sustained relaxation oscillations (ROs). Wieczorek et al.^31^ investigated such an effect in connection with the so-called compost-bomb instability, i.e., an explosive release of soil carbon from peatlands into the atmosphere. Kiers and Jones^32^ and Wieczorek et al.^33^ analyzed conditions for the occurrence of such R-tipping in excitable systems; see also applications of R-tipping to ecosystems^21,23,24^.

Before introducing in more detail the subject of the investigation and the model used, we briefly summarize the concepts of multistability and excitability in climate dynamics. Multistability is one of the main paradigms of climate dynamics^6,16,25^, in which the system possesses multiple equilibria, typically arising via saddle-node or pitchfork bifurcations. Transitions from one equlibrium state to another can occur spontaneously if a threshold is crossed, and subsequent bifurcations can lead to limit cycles, tori and strange attractors. Slow, quasi-adiabatic changes in a parameter often lead to hysteresis between two stable equilibria. The transition from a nearly ice-free climate to a snowball Earth and back^16,34–36^ is just one striking example of such behavior. The tipping induced by parameter drift in multistable climate systems was studied, for example, by Drótos et al.^28^ and by Kaszás et al.^37^.

Another key paradigm of climate dynamics is provided by ROs and their excitability^7,38,39^. ROs are self-sustained if a given threshold is passed, otherwise the system is said to be excitable, in which case ROs can be excited by a stochastic process^40^ through the so-called coherence resonance mechanism^41^. An excitable system need not have multiple equilibria but must have a basic state that can be an equilibrium point, a small-amplitude limit cycle or even a strange attractor occupying a small fraction of the phase space volume visited ultimately by the trajectories. Whatever this basic state, the RO is composed of a rapid, large-amplitude transition that leads the system to an unstable excited state and is followed by a spontaneous, slow return to the original basic state. Relevant examples of paleoclimate and current climate phenomena that have been interpreted in terms of ROs in excitable systems are: the ice ages over the late Pleistocene^42^, the Dansgaard-Oeschger events^43–47^, the Heinrich events^34,48,49^, the multidecadal variability of the Atlantic Meridional Overturning Circulation (AMOC^50–53^), and the interannual variability of the Gulf Stream and Kuroshio Extension^54–61^.

None of the studies above examined the phenomena under consideration from the unifying viewpoint of TPs being crossed due to the gradual change in a key control parameter. Given the previously mentioned pervasive effects of global warming, it is important to shed further light on its potential impact upon the interannual and interdecadal variability of the Kuroshio Extension and AMOC, among other relevant manifestations of intrinsic climate variability.

In this spirit, we study herein the four-variable wind-driven ocean model of Pierini^62^ and systematically investigate its TPs under the action of a smooth drift in the external forcing. This low-order, spectrally truncated quasigeostrophic model is nonlinear and excitable and the TPs mark its transition from an excitable to a self-sustained RO regime. In our analysis, ensemble simulations (ESs) will be carried out to estimate the system’s pullback attractors (PBAs) and to capture therewith its internal variability.

The model was originally developed to study aspects of the Kuroshio Extension dynamics that could not be investigated with the much more realistic models based on the partial differential equations of geophysical fluid dynamics because of the prohibitive computational cost required to do so. In fact, the affordable computational cost of this low-order model did allow stochastic TPs to be studied^63^ and the system’s PBAs to be obtained in several interesting cases^64–67^. Apart from its original oceanographic application, the model should be seen here as a mathematical tool used to investigate basic aspects of excitable systems, and the results so obtained could be helpful in the broader context of the climate sciences and elsewhere.

The main issues addressed in the present study are: (a) finding the forcing amplitude at the TP and its departure from the corresponding frozen-in bifurcation value; (b) the analysis of the dependence of the TP on the forcing’s drift rate; and (c) the sensitivity of the TP to the period and amplitude of periodic perturbations in the forcing.

The paper is organized as follows. In the next section the mathematical model is described, an operational definition of TPs is introduced, and the ES approach is discussed. In the subsequent sections, a basic numerical experiment is presented and analyzed, several sensitivity experiments are discussed and, finally, the results are summarized and conclusions are drawn.

## Model and methods

### The model and the experimental setup

The model used herein describes the wind-driven ocean circulation in midlatitude basins, such as the North Atlantic or North Pacific. In such a circulation, a western boundary current jet, such as the Gulf Stream in the North Atlantic, forms the common boundary of an anticyclonic (i.e., clockwise in the northern hemisphere) subtropical gyre and a cyclonic (i.e., anticlockwise in the northern hemisphere) subpolar gyre^7,26,68^. The analysis here is based on the highly truncated spectral double-gyre model of Pierini^62^. The flow is two-dimensional and it is confined to a rectangular domain; it is described by the streamfunction $\psi ({\mathbf {x}},t)$, with $\psi$ and the cartesian coordinates ${\mathbf {x}} = (x,y)$ being dimensionless. The time *t* in the equations below is also dimensionless but we shall use in the text dimensional time, still denoted by *t*, to emphasize the typical time scales of the oceanic phenomena that have inspired the model, namely the bimodal decadal variability of the Kuroshio Extension^55,69^.

Our quasigeostrophic model is governed by four coupled nonlinear ordinary differential equations, written here in vector–matrix notation as:

1 $$\begin{aligned} \frac{\mathrm {d} {\Psi } }{\mathrm {d} t}+{\Psi } {\mathbf {J}}{\Psi } +{\mathbf {L}}{\Psi } =G\left( t \right) {\mathbf {w}}; \end{aligned}$$

here the vector $\Psi \left( t \right) =\left( \Psi _{1} , \Psi _{2}, \Psi _{3}, \Psi _{4} \right)$ contains the coefficients in the truncated Galerkin expansion of the streamfunction with respect to the orthonormal modes $\{E_i(x,y): i=1, \ldots , 4\}$, with $\psi ( {\mathbf {x}},t ) = \sum _{i=1}^{4}\Psi _{i} ( t ) E_i(x,y)$. Please see Pierini^62^ for the full evolution equation of $\psi ( {\mathbf {x}},t )$, the definition of the matrix operators ${\mathbf {J}}$ and ${\mathbf {L}}$, the wind stress forcing vector ${\mathbf {w}}$, the orthonormal basis $\{E_i(x,y)\}$, as well as for all the technical details and values of the model parameters not mentioned here.

Moreover, Pierini^62^ noticed that $\Psi _{2}$ and $\Psi _{4}$ play a role similar to that of variables that are conjugate to $\Psi _{1}$ and $\Psi _{3}$, respectively; see, for instance, Fig. 4 in Pierini^62^. Hence, the initial data in all the forward time integrations are chosen to satisfy $\Psi _{1}=\Psi _{2}$ and $\Psi _{3}=\Psi _{4}$^64–66^. Moreover, for the sake of convenience, the scaling $10^{-5}\Psi \rightarrow \Psi$ is adopted throughout.

The scalar factor *G*(*t*) in the external forcing of Eq. (1) is chosen as a linear combination of a normalized climatological, time-independent component $\gamma$, a monotonic ramp, and a periodic perturbation:

2 $$\begin{aligned} G( t ) = \gamma +\alpha R_{\tau }( t ) + \beta \sin (\omega t). \end{aligned}$$

**Figure 1:**
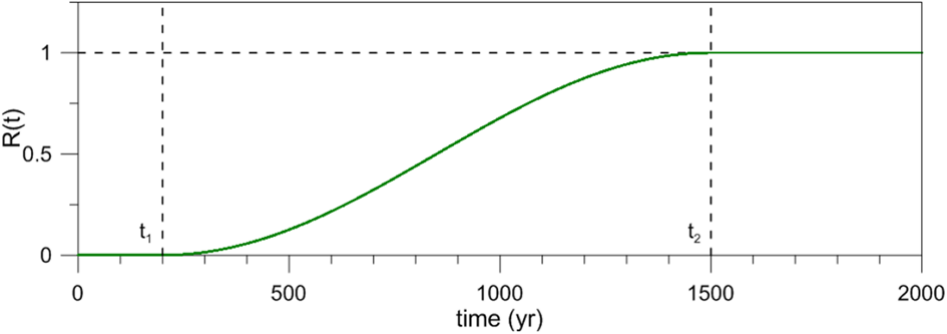
Definition of the ramp function $R_{\tau } ( t )$; its duration is defined as $\tau =t_{2}-t_{1}$.

Here $\gamma$, $\alpha$ and $\beta$ are positive dimensionless constants, $\omega = 2\pi /T$, while $R_{\tau }( t )$ is the ramp function shown in Fig. 1, with $\tau =t_{2}-t_{1}$ and $t_1=200$ year throughout the analysis; the explicit formula for $R_{\tau }( t )$ is given in Supplementary Equation (S1). A similar ramp — with a sigmoid, hyperbolic-tangent shape, rather than the trigonometric shape given by Equation (S1) herein — was used by Daruka and Dietlevsen^70^ in the study of the mid-Pleistocene transition (MPT) in the amplitude and mean period of the Quaternary era’s glacial–interglacial cycles. The MPT and the various approaches used to simulate and explain it are discussed in Section 4 and Appendix A of Riechers et al.^71^

This ramp is approximately linear near its midpoint and it varies smoothly towards the endpoints $t_{1}$ and $t_{2}$. In our analysis, we will characterize the ramp by its steepness $\delta$ calculated at its midpoint:

3 $$\begin{aligned} \delta = \alpha R'_{\tau } |_{t=\left( t_{1}+t_{2} \right) /2}. \end{aligned}$$

Keeping in mind the original application of model (1) to the Kuroshio Extension dynamics, the monotonically increasing component in (2) stands for the effect of amplification in the midlatitude winds due to anthropogenic warming, while the periodic perturbation can be thought of as the seasonal-to-interannual variability in the westerly winds.

The behavior of the model’s autonomous version, in which $\alpha =\beta =0$, is discussed in the Supplementary Information. The critical value $\gamma = \gamma _{\mathrm{c}} = 1$ corresponds to a TP that marks the abrupt transition from small-amplitude limit cycles to large-amplitude, nonlinear, self-sustained ROs; see Supplementary Figures S1, S2 and discussion thereof. In particular, this sudden jump from a small- to a large-amplitude oscillation might be associated with a canard-type transition^72^.

**Figure 2:**
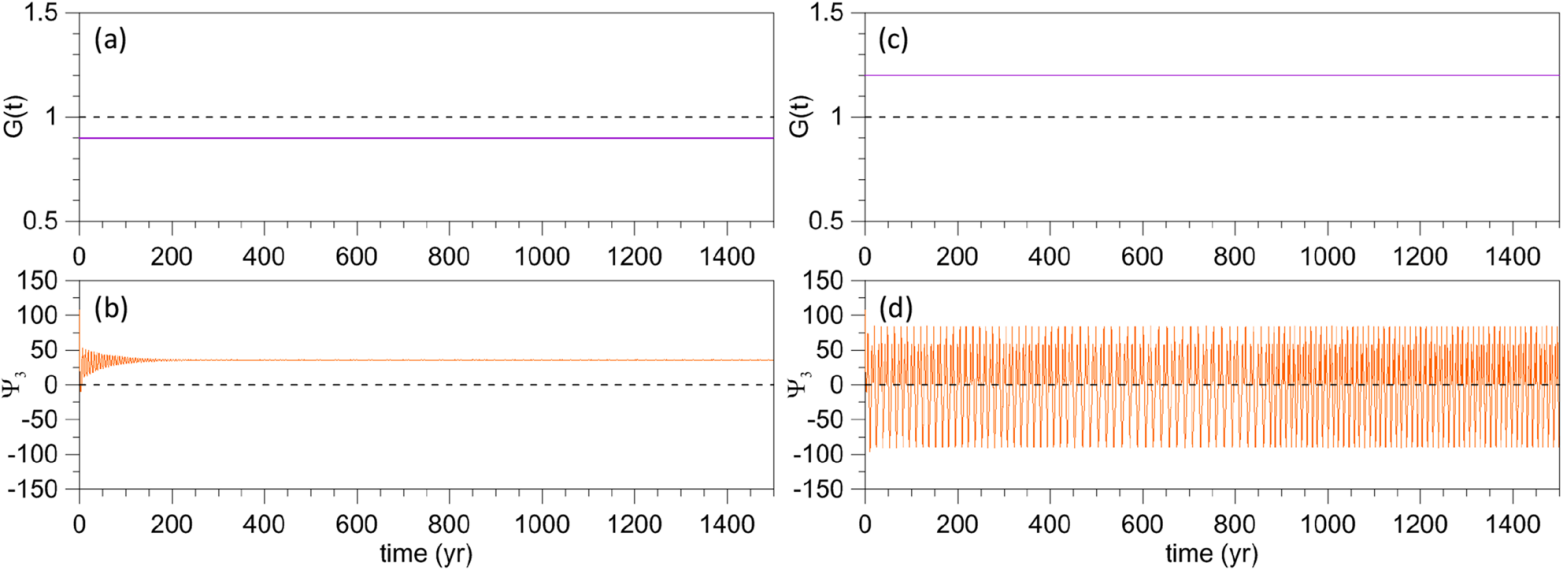
Typical solutions in the two regimes of the model’s autonomous version, cf. Eq. (1), with $\alpha = \beta = 0$ in the forcing given by Eq. (2). The constant values $\gamma$ of the factor *G*(*t*) in the forcing are shown in the upper panels by a solid purple line, with (**a**) $\gamma =0.9$ and (**c**) $\gamma =1.2$. The corresponding model solutions are plotted for $\Psi _{3}(t)$ in the lower panels (**b**) and (**d**); see text and Fig. 4 for the initialization of the trajectories in the lower panels.

As an example of the two types of autonomous behavior, for $\gamma < 1.0$ and $\gamma > 1.0$, Fig. 2b, d illustrate the evolution of $\Psi _{3}(t)$, initialized at the point marked by the red filled circle in Fig. 4 below. A very small-amplitude periodic solution is plotted, for $\gamma = 0.9$, in panel (b) and a large-amplitude RO, for $\gamma = 1.2$, in panel (d) of Fig. 2. In this autonomous case, the value $\gamma _{\mathrm{c}} = 1$ that characterizes transition from small-amplitude oscillations to large-amplitude ROs is identified by the light, black dashed line in panels (a, c).

It is worth stressing that ROs can emerge even for $\gamma < \gamma _{\mathrm{c}}$: a suitable additive noise in *G*(*t*) can excite them and thus activate coherence resonance^62^; hence, $\gamma _{c} = 1$ identifies the upper bound of the so-called excitable regime. Pierini^63^ introduced a stochastic TP $\gamma _{\mathrm{s}}$ to define the lower bound of this regime. A similar phenomenon was described by Sutera^73^ in the Lorenz convection model^3^, where it is associated with the existence of a subcritical Hopf bifurcation; see Sec. 5.4 in ref.^6^ and Fig. 5.9 therein. In this earlier work, the role of the unstable RO here is played by an unstable limit cycle.

In Supplementary Figure S3 and the related discussion, it is shown that our model possesses the fundamental property of excitability when subjected to noisy forcing. This property is common to the excitable systems relevant to the climate sciences mentioned in the introduction, ranging from paleoclimatic^34,42–49^ to multidecadal^50–53^ and down to interannual time scales^54–61^.

### Tipping point definition for time-dependent forcing

**Figure 3:**
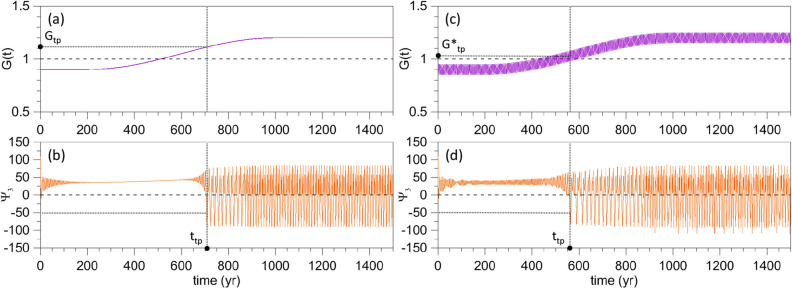
Transition from the excitable regime to the RO regime for a ramp forcing in Eq. (2). (**a**) Time dependence of the factor *G*(*t*) in the forcing for $\gamma = 0.9 < \gamma _{\mathrm{c}} = 1$, $\alpha =0.3$, $\tau =800$ year, and $\beta =0$; (**b**) corresponding response of $\Psi _{3}(t)$; see text for the choice of initial states. (**c**, **d**) Same as panels (**a**, **b**) but for $\beta = 0.05$ and perturbation period $T=5$ year.

The question we want to answer in the present study is: how does the transition from small-amplitude limit cycles to large-amplitude ROs occur if *G*(*t*) is increased gradually from a subcritical value $G < \gamma _{\mathrm{c}} = 1$ to a supercritical value $G > 1$? More specifically, at what time and for what value of *G* does the abrupt transition occur? Figure 3 helps clarify this question.

Figure 3a shows the time dependence of *G*(*t*) for $\gamma = 0.9$. Thus, *G* passes gradually from its value in Fig. 2a to that in Fig. 2c. The corresponding response is shown in Fig. 3b: the transition does not occur for $G = 1$, as one might expect it; such a transition would require an infinitely slow increase of *G*(*t*), so as to take the system from the excitable regime to the RO regime adiabatically through a sequence of quasi-autonomous states. In fact, if we choose to identify the transition time—denoted here as the TP time $t_{\mathrm{{tp}}}$—as the one at which $\Psi _{3}$ is less than the threshold value $\Psi _{3,\mathrm{c}} = -50$ for the first time, then the transition takes place at $t = t_{\mathrm{{tp}}} = 707$ year, which corresponds to $G(t_{\mathrm{{tp}}})\equiv G_{t_{\mathrm{{tp}}}} = 1.11$ and is appreciably greater than $\gamma _{\mathrm{c}} = 1$, cf. Fig. 3a, b.

One might then wonder how robust this TP is. The simplest way to address this question is to add a sinusoidal perturbation to the ramp. In this case, i.e., if $\beta \ne 0$, we will use

4 $$\begin{aligned} G^{*}(t_{\mathrm{{tp}}})\equiv G^{*}_{\mathrm{{tp}}} =\gamma +\alpha R_{\tau }(t_{\mathrm{{tp}}}) \end{aligned}$$

to characterize the forcing amplitude at the TP. The example plotted in Fig. 3c, d differs from that of Fig. 3a, b only by the presence of such a perturbation in Eq. (2), with $\beta =0.05$ and $T=5$ year. As a result, $t_{\mathrm{{tp}}}$ occurs earlier, at $t_{\mathrm{{tp}}} = 563.5 < 707$ year, whereas the amplitude is reduced to $G^{*}_{\mathrm{{tp}}} = 1.029 < 1.11$.

The methodology illustrated in Figs. 2 and 3 will be followed throughout our analysis, but the ESs mentioned in the introduction will be used to capture the system’s internal variability, as described in the next section.

### Ensemble simulations

For each time-dependent forcing *G*(*t*) used in this study, a 1500-year-long ES composed of $N=168$ members will be carried out: this will provide an estimate of the system’s PBA. The initial points corresponding to the ensemble members will be regularly distributed at $t=0$ in the four-dimensional hypercube $\Omega \equiv \{\ - 70 \le \Psi _{1},\Psi _{2} \le 150 ;\quad -150 \le \Psi _{3},\Psi _{4} \le 120 \}$. For the sake of graphical representation,we will refer to the rectangle $\Gamma \equiv \{\ - 70 \le \Psi _{1} \le 150; \quad -150 \le \Psi _{3} \le 120\}\subset \Omega$ lying in the $(\Psi _{1},\Psi _{3})$-plane, as done in Pierini^64^ and in subsequent analyses of model (1). Moreover, in the discussion of the results, we will refer to the model’s trajectories as being defined in the $(\Psi _{1},\Psi _{3})$-plane but, naturally, the actual trajectories evolve in the full four-dimensional phase space.

**Figure 4:**
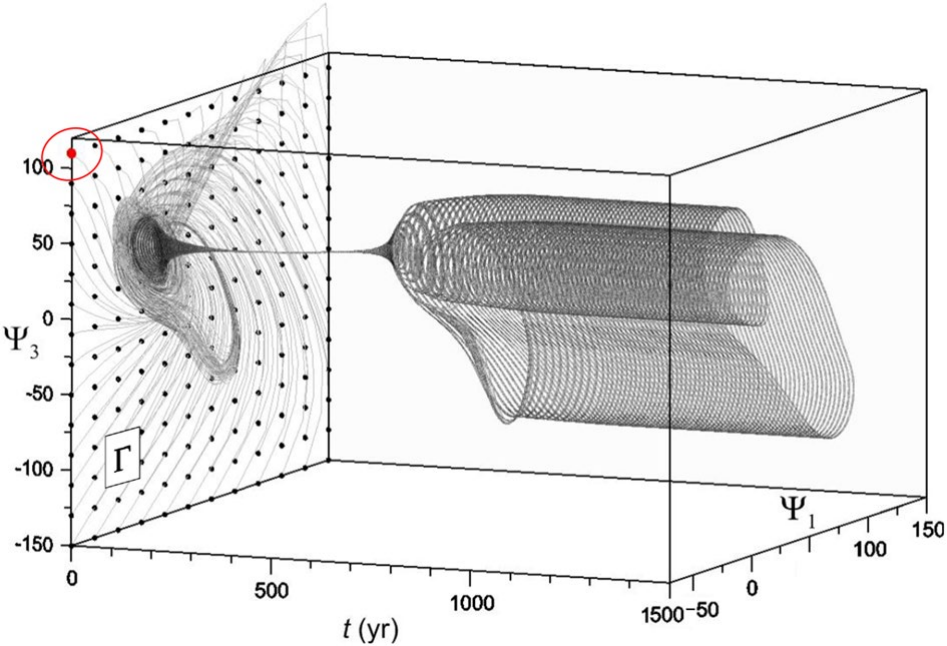
ES subject to the same forcing used in the single forward time integrations of Fig. 3a, b. The filled red circle in the oval indicates the initial state used to initialize the four simulations of Figs. 2 and 3. After an initial transient, it is visually obvious that the ES converges to a stable cylinder-shaped PBA, obtained by the translation in time of an autonomous limit cycle.

Figure 4 shows the ESs corresponding to the simulation of Fig. 3a, b, whose single integration is initialized at the red point in the upper-left corner of $\Gamma$; the regular grid in $\Gamma$ indicates the 168 initial points for the ES. The definition of a TP for the time-dependent forcing introduced above is extended to an ES as follows: $t_{\mathrm{{tp}}}$ is the time at which, for the first time in any of the ES members, $\Psi _{3} < \Psi _{3, \mathrm{c}}$.

## Results: the basic numerical experiment

In this section, a numerical experiment composed of 80 ESs is presented and discussed; it is denoted as Exp1 in Table 1 and it serves to study the TP’s dependence on the ramp steepness $\delta$. For this study, we let $\tau$ take on 80 distinct values that range from very abrupt change in the forcing, with $\tau = 32.5$ year, to very gradual, with $\tau = 1316.25$ year. Except for $\tau$, all the other forcing parameter values in Eq. (2) here are the same as used for the simulations in Figs. 3a, b and 4, namely $\gamma =0.9$, $\alpha =0.3$ and $\beta =0$. Moreover, for each $\tau$-value, an ES with 168 initial data—such as that shown in Fig. 4—is carried out in order to simulate the irreducible uncertainty associated with the system’s internal variability^74–76^. The ramps in *G*(*t*) for the two extreme values of $\tau$ are plotted in Supplementary Figure S4.

**Table 1:** List of the numerical experiments; see Eq. (2) for the definition of the parameters. In experiments Exp1–Exp6, the duration $\tau = t_2 - t_1$ of the ramp takes on 80 different values that range from 32.5 to 1316.25 year. In experiments Exp7 and Exp8, the period *T* ranges from 1 to 100 year. For each value of $\tau$ or *T*, an ES with 168 initial states is carried out. Quotation marks in the table indicate identical entries.

Numerical experiment	$\gamma$	$\alpha$	$\tau$ (year)	$\beta$	*T* (year)
Exp1	0.9	0.3	32.5–1316.25	0	−
Exp2	0.8	0.4	”	”	−
Exp3	0.9	0.3	”	0.025	5
Exp4	”	”	”	0.050	”
Exp5	0.8	0.4	”	0.025	”
Exp6	”	”	”	0.050	”
Exp7	0.9	0.3	800	0.050	1–100
Exp8	”	”	”	0.100	”

**Figure 5:**
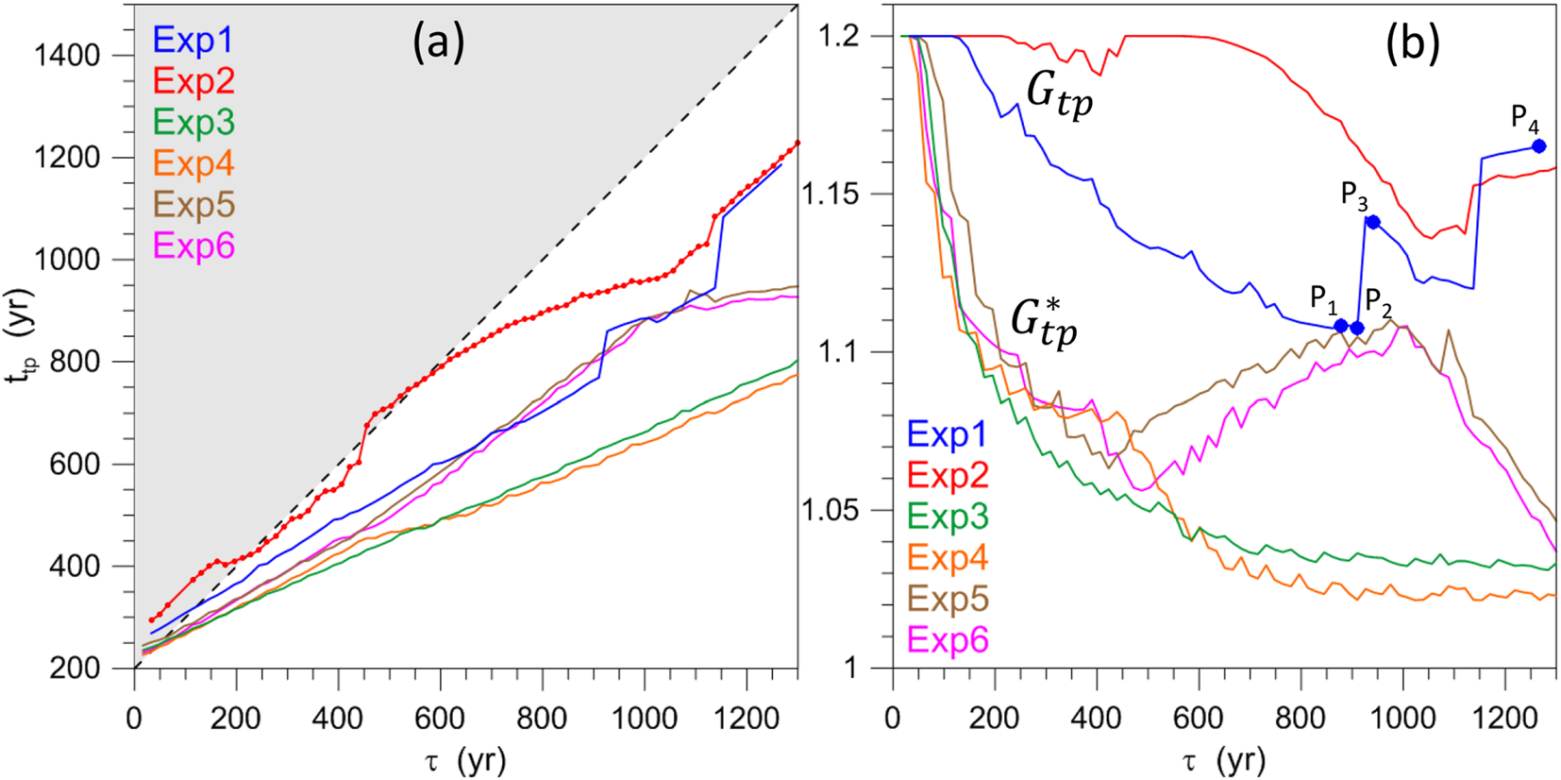
Dependence of key results on the ramp duration $\tau$ in experiments Exp1–Exp6. (**a**) TP timing $t_{\mathrm{{tp}}}$ versus $\tau$; when a line portion lies in the grey area the tipping occurs after the end of the ramp. The filled circles on the red line indicate the presence of data; their absence indicates that no TP is reached. (**b**) Forcing values $G(t_{\mathrm{{tp}}})$ at the TP (for Exp1 and Exp2) or $G^{*}_{\mathrm{{tp}}}$ (for Exp3–Exp6) versus $\tau$; the filled blue circles $P_1{-}P_3$ correspond to the ESs for Exp1 shown in Supplementary Figure S5, while the filled circle $P_{4}$ corresponds to the orange ES of Supplementary Figure S6.

Figure 5 summarizes the results of Exp1–Exp6, but we only discuss here those of Exp1; the results of Exp2–Exp6 will be discussed in the following section. The blue line in Fig. 5a shows the dependence of $t_{\mathrm{{tp}}}$ on $\tau$ in Exp1: $t_{\mathrm{{tp}}}$ increases monotonically and almost linearly with $\tau$; the anomalous jumps and fluctuations appearing after $t \simeq 1100$ year will be explained below. The monotonically increasing trend is obviously due to the fact that the greater $\tau$ the longer it takes to reach a given value of *G*.

The blue line in Fig. 5b shows that $G_{\mathrm{{tp}}}$ decreases almost linearly up to $\tau \cong 900$ year as $\tau$ increases. Such a negative trend is also expected because, as already observed, the slower the variation of the forcing strength the closer the forcing at the TP will be to the autonomous critical value $G=1$, i.e., we expect $G_{\mathrm{{tp}}} \rightarrow 1$ as $\tau \rightarrow \infty$.

It is important, though, to stress that the range of variability of the ramp’s duration $\tau$ considered herein spans time intervals that range from roughly 3 to roughly 130 times the typical time scale of the ROs; hence $G_{\mathrm{{tp}}}$ is always substantially greater than unity. Note that the initial plateau for $\tau \le 130$ year in Fig. 5b is present because for those values the TP is reached at $t > t_{2}$, i.e., when $G=1.2$.

Thus, in the range 130 year $< \tau < 900$ year, as $\tau$ increases—and therefore the forcing’s drift rate $\delta$ decreases—the TP is delayed and the corresponding *G*-value decreases, but it remains well above the autonomous critical value $G=1$. For $\tau > 900$ year, a sudden increase of $G_{\mathrm{{tp}}}$ occurs in Fig. 5b, followed by a gradual decrease and another sharp increase. We investigate these anomalous behaviors in the Supplementary Information (see Supplementary Figures S5, S6 and S7).

**Figure 6:**
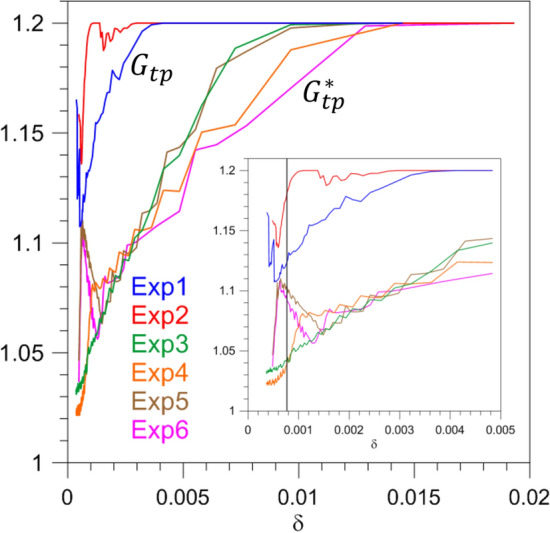
Same as Fig. 5b but shown as a function of the ramp steepness $\delta$ defined in Eq. (3). The solid black vertical line in the zoomed inset indicates the ramp steepness $\delta$ corresponding to the two ESs of Exp1 (blue line) and Exp2 (red line) shown in Fig. 8.

According to Fig. 5a, as $\tau$ increases, the TP is delayed and the corresponding value of $G_{\mathrm{{tp}}}$ in Fig. 5b decreases. It follows that, since increasing $\tau$ the ramp steepness $\delta$ decreases, $G_{\mathrm{{tp}}}$ must increase with $\delta$. This increase is shown in Fig. 6 for Exp1 by the blue line: the forcing amplitude $G_{\mathrm{{tp}}}$ at the TP increases steeply and monotonically with the drift rate, except for the anomalous behavior found for $\delta <0.0005$ and $\delta >0.004$. The latter nonmonotonicities are associated with the appearance and disappearance of local PBAs (see the Supplementary Information).

To complete the analysis of Exp1 we investigate the sensitivity to initial data of the ES members. Supplementary Figures S5, S6 and S7 show that the RO phases tend to cluster in groups or may even exhibit total independence from the initial data (TIID), as occurs in Supplementary Figure S5(b) for some of the *N* trajectories. To obtain information about the phase dependence of the ensemble members, we rely on the parameter *C* introduced by Pierini^64^:

5 $$\begin{aligned} C( r,t,\tau ) = \frac{1}{N^{2}}\sum _{i,j}^{N}H\left[ r-\left| {\Psi }_{\tau }^{( i )}( t ) - {\Psi }_{\tau }^{( j )}( t ) \right| \right] ; \end{aligned}$$

here *H* is the Heaviside step function and *r* is a prescribed distance. This functional *C* gives the number of trajectory pairs whose distance is less than *r*, normalized by $N^{2}$. In general, if the *N* available trajectories reduce to *n* clusters, each one containing trajectories within the maximum distance *r*, then $1/n\le C\le 1$. In the present analysis each of these clusters will be denoted as “single trajectory” if $r \le r_{0}=0.5$; $r_{0}$ is in fact much smaller than the projection of the PBA onto the $\Gamma$-plane. Note that the extreme case $C=1/n$ occurs if the *N* trajectories are equally distributed among *n* single trajectories.

The particularly interesting case $C=1$ ($n=1$) implies TIID and is usually referred to as *generalized synchronization* when the forcing is periodic, because then the unique single trajectory is necessarily synchronized with the forcing (e.g.,^64,77,78^). More generally, the existence of a small number of single trajectories is clearly a case of phase synchronization^79–81^.

A time-independent parameter can be obtained by averaging *C* over an interval $T_{0}$ starting from the tipping time $t_{\mathrm{{tp}}}$:

6 $$\begin{aligned} {\bar{C}}( r,\tau )=\frac{1}{T_{0}}\int _{t_{\mathrm{{tp}}}( \tau )}^{t_{\mathrm{{tp}}}\left( \tau \right) +T_{0}}C ( r,t,\tau )dt. \end{aligned}$$

**Figure 7:**
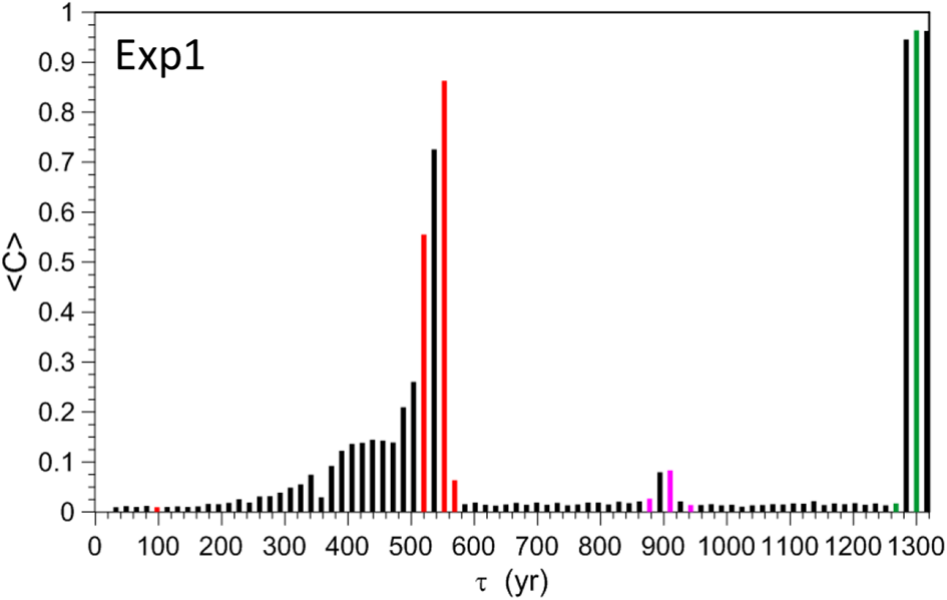
Dependence of the number ${\bar{C}}$ of trajectory clusters on $\tau$ for Exp1, with $T_{0}=50$ year and $r=0.5$ in Eq. (6). The magenta, green and red bars refer to the ESs shown in Supplementary Figures S5, S6 and S8, respectively.

Figure 7 shows ${\bar{C}}\left( r,\tau \right)$ with $r= r_{0}= 0.5$ and $T_{0}=50$ year. See Supplementary Figure S8 for a discussion of the four red bars. As $\tau$ is increased further in Fig. 7, the ${\bar{C}}$-values for the ESs reported in Supplementary Figures S5 and S6 are plotted as the magenta and green bars, respectively. In particular, the unstable trajectories corresponding to $\tau =1300$ year (cyan lines in Supplementary Figures S6 and S7(b)) yield a value of ${\bar{C}}=0.96$ close to TIID, as expected.

## Results: sensitivity experiments

In this section, we study the experiments Exp2–Exp8 summarized in Table 1, focusing on the changes in TPs induced by the changes in the parameters $\gamma , \alpha , \beta$ and $\tau$ of the forcing in Eq. (2).

### Sensitivity of tipping to the system’s past history (Exp2)

In Exp1, we saw that the TP depends crucially on the ramp steepness $\delta$ of the forcing *G*(*t*), which one can also think of as the drift rate; see again Figs. 5 and 6. Ashwin et al.^18^ defined rate-induced tipping for systems possessing multiple steady states^16,82^; the blue line of Fig. 6 illustrates a similar phenomenon occurring in our excitable system for Exp1.

However, a natural question arises: does the TP depend solely on $\delta$, as the R-tipping terminology suggests, or does it dependent also—and perhaps crucially—on other parameters, too? To clarify this issue, we designed and performed Exp2: it differs from Exp1 in that the climatological amplitude in Eq. (2) is reduced from $\gamma =0.9$ to $\gamma =0.8$, while the ramp factor is increased from $\alpha =0.3$ to $\alpha =0.4$. As a consequence, Exp1 and Exp2 share the same $G_{max}$ while, for a given $\tau$, $\delta$ is larger in Exp2 then in Exp1. We will therefore be able to compare ESs with the same $\delta$ but with a different past history.

**Figure 8:**
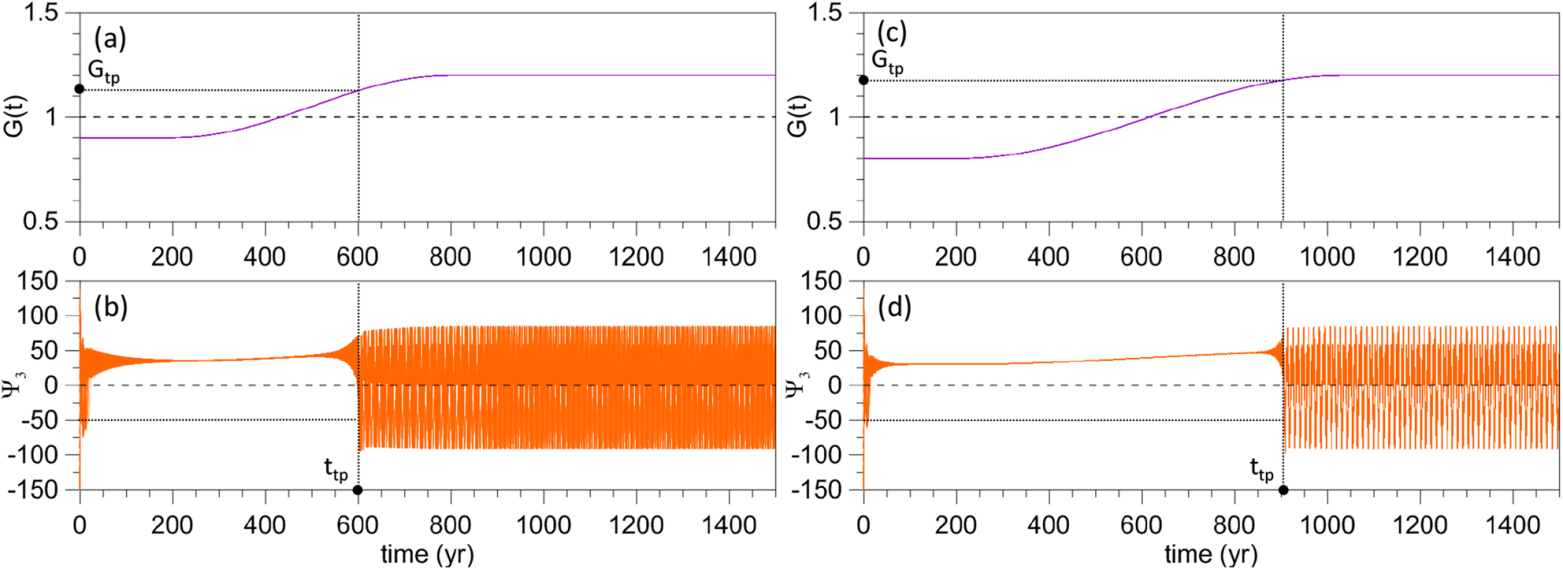
Dependence of the TP on ramp length for two ESs belonging to Exp1 and Exp2 and having the same drift rate $\delta$. (**a**) Time dependence of the factor *G*(*t*) for Exp1 with $\tau = 600$ year. (**b**) $\Psi _{3}(t)$ of the corresponding ES. (**c**) Time dependence of the factor *G*(*t*) for Exp2 with $\tau = 835$ year. (**d**) $\Psi _{3}(t)$ of the corresponding ES.

In Fig. 8, the details of an ES that is part of Exp1 are thus compared with those of an ES within Exp2. The two are chosen so that $\delta$ is the same in both, as shown by the vertical solid line in the zoomed inset of Fig. 6, but their past histories differ. In Exp2, both $t_{\mathrm{{tp}}}$ and $G_{\mathrm{{tp}}}$ are found to be greater than in Exp1. More generally, the red solid lines of Exp2 are quite distinct from, and lie well above, the blue solid lines for Exp1 in both Figs. 5 and 6, where the dependence of $t_{\mathrm{{tp}}}$ and $G_{\mathrm{{tp}}}$ on $\tau$ and $\delta$, respectively, is plotted.

Thus, in the case of forcing that increases monotonically, the transition is not determined by the drift rate $\delta$ alone but by both $\delta$ and the initial forcing amplitude. More precisely, based on the comparison between the dependence of $G_{\mathrm{{tp}}}$ on past history at given $\delta$ for Exp1 and Exp2, as shown in Fig. 6, we can conclude that the smaller the forcing amplitude $\gamma <1$ at the beginning of the ramp, the greater the forcing amplitude at the TP has to be.

This result clearly implies a long memory of the past. For example, let us consider the ESs of Exp1 and Exp2 shown in Fig. 8. Suppose that, in Exp1, at $t=t_{a}$ the model has not yet reached the TP for $G(t_{a})=G_{a}$; then suppose that, in Exp2, at $t=t_{b}$ the model has not yet reached the TP either for the same $G(t_{b})=G_{b}=G_{a}$. Still, Fig. 8b, d show that, shortly before the TP, the state of the system is virtually the same in both ESs.

In summary, at $t=t_{a}$ in Exp1 and at $t=t_{b}$ in Exp2, the state of the system is virtually the same, $\delta$ is the same, while the value of the external forcing and its future evolution are the same as well. Yet the TP is reached at very different times with respect to the beginning $t_1$ of the changes in the forcing. Since the only difference between Exp1 and Exp2 is the temporal evolution prior to $t_{a}$ and $t_{b}$, respectively, this effect can only occur if the system keeps track of the previous evolution for sufficiently long times. One might expect that, if the system is perturbed, such an anomalous behavior would no longer occur.

We will see in the next subsection that, in fact, if the forcing is perturbed by a periodic component, the scenario just discussed changes drastically. Namely, over a wide range of $\delta$-values, the TP depends solely on $\delta$. The latter behavior represents, therefore, a case of R-tipping in an excitable system.

Finally, notice that, unlike in Exp1, ${\bar{C}} \simeq 1$ in Exp2, and thus the latter exhibits TIID for many more values of $\tau$, as seen by comparing Supplementary Figure S9 with Fig. 7. An example is provided by the ES of Fig. 8d, in which the *N* ensemble members are well synchronized. The reason for such a difference in behavior deserves to be analyzed in detail in a future study. In the next subsection, we will at least investigate whether the TIID observed in Exp2 is stable or not.

### Sensitivity to periodic perturbations (Exp3–Exp6)

It is important to assess the robustness of the results obtained in Exp1 and Exp2 with respect to perturbations of the forcing. We saw that a small-amplitude periodic perturbation added to the forcing can affect considerably $t_{\mathrm{{tp}}}$ and $G^{*}_{\mathrm{{tp}}}$, cf. Figs. 3c, d, while Supplementary Figure S7 shows that even when such a periodic perturbation acts only over a short duration it may trigger an instability in the solution. Therefore, we now repeat Exp1 and Exp2 by adding periodic perturbations in *G*, with $\beta \ne 0$ in Eq. (2). The perturbation period is 5 year and we use two amplitudes, $\beta =0.025$ and 0.05. Exp3 and Exp4 correspond to Exp1, while Exp5 and Exp6 correspond to Exp2; see Table 1.

Compare now in Fig. 5 (1) the curve of Exp1 (blue) with those of Exp3 (green) and Exp4 (orange) and (2) that of Exp2 (red) with those of Exp5 (brown) and Exp6 (magenta). From panel (a) it is immediately clear that, as a result of the perturbation, $t_{\mathrm{{tp}}}$ occurs notably earlier, while panel (b) clearly shows that the forcing amplitude $G^{*}_{\mathrm{{tp}}}$ is notably reduced. Note also that the difference (1) between Exp3 and Exp4 and (2) between Exp5 and Exp6 is quite small, despite their perturbation amplitudes differing by a factor of 2.

These two findings suggest that the TPs that are due to a drift in the forcing, as in Exp1 and Exp2, are unstable: a small periodic perturbation shifts the TPs to values that are then robust with respect to changes in the perturbation’s amplitude. This result will be analyzed in greater depth in a future study and we strongly suspect that similar sensitivity to random, rather than periodic, perturbations will be also detected.

Another significant example of the instability of the unperturbed Exp1 and Exp2 concerns the dependence of $G_{\mathrm{{tp}}}$ on $\delta$. We saw big differences between Exp1 and Exp2 (blue and red lines in Fig. 6) in this dependence. On the other hand, we argued there that, since such differences seem to be associated with the system’s full history, the same value $G^{*}_{tp}(\delta )$ could result if the two histories of the forcing were subjected to a periodic perturbation.

**Figure 9:**
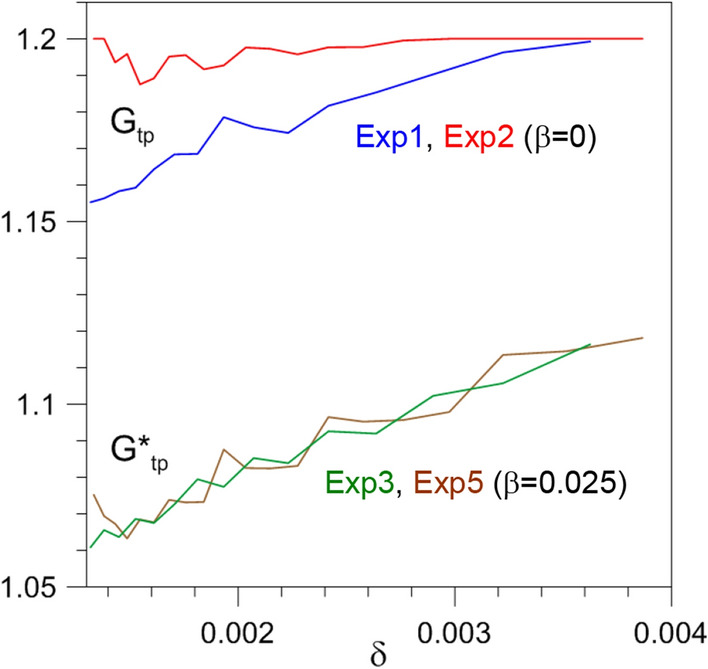
Rate-induced tipping in the presence of periodic perturbations. Same as Fig. 6 but for a limited range of $\delta$-values for the unperturbed (Exp1 and Exp2) and corresponding perturbed (Exp3 and Exp5) cases with $\beta =0.025$ and $T=5$ year.

This is in fact what occurs for a significant range of $\delta$-values. Let us focus on $\delta \ge 0.0013$ in Fig. 6, which is beyond the range of anomalous behaviors discussed in connection with the basic numerical experiment. The lines showing $G_{\mathrm{{tp}}}(\delta )$ for the unperturbed Exp1 and Exp2 and the lines showing $G^{*}_{tp}(\delta )$ for the corresponding perturbed Exp3 and Exp5 with $\beta =0.025$ are again reported, for $0.0013 \le \delta \le 0.004,$ in Fig. 9. For the sake of clarity, Exp4 and Exp6, with $\beta =0.05$, are not plotted, since they yield basically the same behavior.

The first striking difference between the unperturbed and perturbed cases concerns the substantially smaller values attained by the forcing amplitude at the TP for the perturbed cases, as just discussed. For values of the drift rate approaching $\delta \simeq 0.004$, the blue and red lines of Exp1 and Exp 2 both tend to the constant value $G_{\mathrm{{tp}}} = 1.2$ because, as already noted, in this limit the TP is reached at $t > t_2$ when $G=1.2$. For decreasing $\delta$ the difference between the two curves increases, as already discussed.

On the contrary, the lines (green and brown) showing $G^{*}_{\mathrm{{tp}}}(\delta )$ for Exp3 and Exp5 essentially coincide in this range. The memory of the remote past states of the perturbed system is lost, and the same forcing amplitude at the TP is reached in Exp3 and Exp5—which, like Exp1 and Exp2, share the same mean drift rate. In conclusion, the green and brown lines of Fig. 9 show a case of R-tipping in an excitable system.

In the Supplementary Information, interesting results are presented concerning the dependence of the phase distribution of ROs on the presence and amplitude of periodic forcing (see Supplementary Figures S10 and S11).

### Sensitivity to forcing period (Exp7 and Exp8)

**Figure 10:**
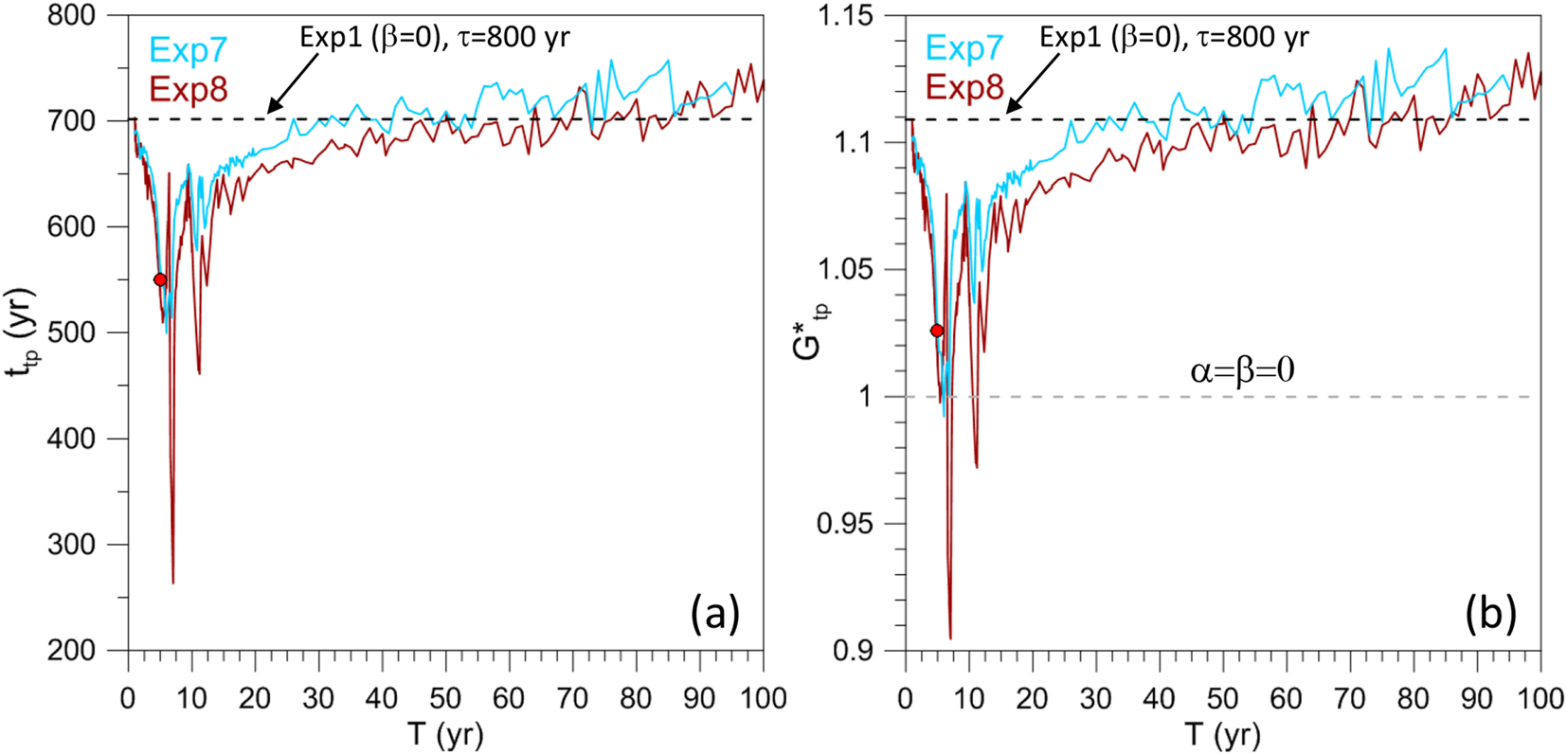
Dependence of the TP on period *T* in Exp7 and Exp8. (**a**) TP timing $t_{\mathrm{{tp}}}$ versus period *T*. The horizontal dashed line indicates the value corresponding to Exp1 for $\tau = 800$ year. (**b**) $G^{*}_{\mathrm{{tp}}}$ versus *T*. Black dashed line as in panel (**a**), while the gray dashed line corresponds to the critical value of the autonomous system. The red filled circles in both (**a**) and (**b**) indicate the value that is common to Exp7 and Exp4.

In the preceding subsection, we have studied the effect of a periodic perturbation of fixed period $T=5$ year on the TPs induced by a drift in the forcing for varying $\tau$. In order to analyze the sensitivity to forcing period, Exp1 is modified in Exp7 and Exp8 to include a periodic forcing in which $\tau = 800$ year is fixed, while the period *T* varies from 1 to 100 year; see again Table 1. The dependence of the TP on *T* in these two numerical experiments is shown in Fig. 10. Like in the previous numerical experiments we compute 80 ESs—in this case with *T*-values regularly distributed in the range 1–100 year—to construct the graphs of Fig. 10. Many additional ESs are also computed for $T=1$–25 year so as to capture the strong variability of $t_{\mathrm{{tp}}}$ and $G^{*}_{\mathrm{{tp}}}$ in this range.

Let us first focus on Exp7 (light blue lines), which shares the forcing amplitude $\beta =0.05$ with Exp4. For high values of *T*, ranging between 25 and 100 year, $t_{\mathrm{{tp}}}$ and $G^{*}_{\mathrm{{tp}}}$ are close to the corresponding value of Exp1, with no periodically perturbed forcing (black dashed line). For $T\simeq 10$–25 year the TP occurs somewhat earlier, while for $T<10$ year abrupt drops in $t_{\mathrm{{tp}}}$ do occur. The biggest drop is at $T \cong 6$ year and it corresponds to an earlier occurrence of the TP by about 200 year with respect to the periodically unperturbed case. For even smaller *T*, $t_{\mathrm{{tp}}}$ and $G^{*}_{\mathrm{{tp}}}$ return to their unperturbed values, while passing through the values that correspond to Exp4 at $T=5$ year; the latter values are indicated by a red filled circle.

A similarly anomalous behavior is found in Exp8 for the perturbation’s amplitude $\beta$ being doubled (brown lines in Fig. 10); in this case the sensitive dependence on the period *T* is even more striking. Chekroun et al.^83^ studied such a rough parameter dependence in a truncated version of a periodically driven, intermediate coupled ENSO model^84,85^ by using the Ruelle–Pollicott resonances of the model’s associated Perron–Frobenius or transfer operator^86,87^.

In the ENSO case of Chekroun et al.^83^, the period of the forcing was fixed and given by the seasonal cycle, while the parameter $\delta$ of interest affected the travel time of the equatorially trapped waves and hence the intrinsic model periodicity. On the other hand, in the present case the intrinsic period of the ROs is determined by the system’s dynamics, while the external-forcing period is being modified. But it is really the ratio of the external-to-internal frequency that matters, as illustrated in the highly simplified case of Arnold tongues in the standard circle map^88,89^.

Returning to Fig. 10 (brown line), two peaks at $T\cong 7$ year and $T\cong 11.2$ year are found in Exp8 and, in both cases, $G^{*}_{tp} < 1$: that is, the ROs arise for a time-averaged forcing amplitude that is appreciably smaller than the value required for the autonomous system to transit from the excitable to the self-sustained RO regime.

The abrupt reduction of $t_{\mathrm{{tp}}}$ and $G^{*}_{\mathrm{{tp}}}$ occurs for periods *T* that are comparable to the RO’s typical time scale; thus, we are in the presence of a nonlinear-resonance–like behavior. These results deserve further investigation by relying on some of the tools discussed in connection with the ENSO case above.

## Summary and conclusions

Excitable dynamical systems are characterized by large-amplitude relaxation oscillations (ROs), which are self-sustained once a control parameter exceeds a given threshold, given by $\gamma =1$ in our case. In such a setting, the system leaves a basic state—e.g., a small-amplitude limit cycle—visits one or more very distinct states, and then returns spontaneously to the basic state. Alternatively, if the control parameter is below that threshold, ROs can be excited by a suitable time-dependent external forcing, in which case the ROs are very similar to those arising in the self-sustained regime. The excitable-system paradigm plays an important role in climate dynamics in general and in paleoclimatology in particular, as discussed in the introduction.

In this paper, we have studied the transition from the excitable to the self-sustained regime subject to the action of a smooth parameter drift. If the drift is infinitely slow, the transition will occur at the same threshold as for the corresponding autonomous system, but for finite drift times the tipping point (TP) marking such a transition could be very different. Investigating this problem is, for example, very important for understanding how internal modes of climate variability could undergo abrupt transitions in amplitude or character as a consequence of the present smooth increase of atmospheric greenhouse gas concentrations^9,16,74^.

We have explored this problem herein by making use of a low-order quasigeostrophic model^62^ originally developed to study the wind-driven ocean circulation; see Eq. (1). The model was used in the present paper as a prototype of an excitable system, given its RO dynamics and its excitability. The various forms of the forcing were given by Eqs. (2)–(3).

In the basic numerical experiment Exp1, the subcritical climatological amplitude $\gamma =0.9$ was connected to the supercritical value $\gamma =1.2$ through 80 ramps differing by their duration $\tau$. The corresponding ramp steepness $\delta$ varies essentially in inverse proportion to $\tau$, cf. Eq. (3). For each ramp, an ensemble simulation (ES) was performed to obtain an approximate description of the corresponding pullback attractor (PBA).

Note that Pierini et al.^65^ rigorously demonstrated the existence of a global PBA for the weakly dissipative nonlinear model governed by Eq. (1). The ESs carried out in the present paper provide us with much more detailed information on the irreducible uncertainty associated with this excitable system’s internal variability.

We performed a detailed analysis of the time $t_{\mathrm{{tp}}}$ at which tipping occurs and the ROs arise, as well as of the corresponding forcing amplitude $G_{\mathrm{{tp}}}$, as a function of $\tau$. The results were summarized in Fig. 5 and in the companion Fig. 6, in which $G_{\mathrm{{tp}}}$—or $G^{*}_{\mathrm{{tp}}}$ for the periodically perturbed cases, with $\beta \ne 0$ in Eq. (2)—is plotted against the ramp steepness $\delta$.

The main result is that—as $\tau$ increases, and therefore $\delta$ decreases—$t_{\mathrm{{tp}}}$ is delayed and $G_{\mathrm{{tp}}}$ decreases, while remaining well above the autonomous critical value $G=\gamma _{c} = 1$. There are, however, important departures from this behavior. For example, in an anomalous dependence of $G_{tp}$ upon $\tau$, two clusters suddenly appear in the PBA and, for a small increase of $\tau$, the first cluster disappears, leading to an abrupt forward shift of the TP; see again Supplementary Figure S5. Besides, we found PBAs for which total independence from the initial data (TIID) occurs and no ROs appear; such filamentary PBAs, however, were shown to be unstable, cf. Supplementary Figure S7.

Rate-induced tipping or R-tipping (e.g.,^18^) has been extensively studied in the literature for systems possessing multiple steady states^16,22,24,82^. The latter is, however, not the case of system (1) herein. Still, in our Exp1, we saw that, in fact, the tipping does depend on the forcing’s drift rate $\delta$ (blue line of Fig. 6).

To investigate whether other conditions contribute to the tipping besides the drift rate, we studied Exp2 to compare ESs with the same $\delta$ but with different forcing histories. Figure 6 shows that the tipping does depend crucially on the temporal evolution of the forcing prior to the autonomous critical value $G_{tp}=1$ being attained. This implies a long memory of the system’s forcing history under certain circumstances. We found, though, that when the forcing is periodically perturbed (i.e., when $\beta \ne 0$ in Eq. (2)) tipping induced solely by the drift rate—that is, R-tipping—does occur, as found for an extended range of drift rates.

Other interesting features were found in Exp2. Unlike in Exp1, TIID behavior is present for many values of $\tau$, as shown in Supplementary Figure S9 in terms of the clustering parameter ${\bar{C}}$ defined in Eqs. (5) and (6). This prevalence of TIID behavior suggests that approaching the TP from smaller initial values of the forcing amplitude *G*(*t*) and with a larger $\delta$ facilitates the appearance of phase coherence. Disjoint local PBAs also coexist for some ramp steepness values; see, for instance, Supplementary Figure S11 (panels (a, b)). Moreover, for some ESs in Exp2, the TP is reached only well after the forcing amplitude has achieved a constant value, cf. Supplementary Figure S11 (panels (a–d)).

In the four numerical experiments Exp3–Exp6, we have investigated how robust the results obtained so far are with respect to small-amplitude periodic perturbations superimposed on the ramp; see again Table 1. As a consequence of these perturbations, $t_{\mathrm{{tp}}}$ occurs noticeably earlier and the forcing amplitude $G^{*}_{\mathrm{{tp}}}$ at the TP is substantially reduced, cf. Figs. 5 and 6. On the other hand, the amplitude $\beta$ of the periodic perturbation does not seem to play a decisive role: doubling $\beta$ from a value of 0.025 to 0.050 does not affect the TP substantially.

Other intriguing features emerge in these periodically perturbed numerical experiments: (1) the disjoint PBAs and the filamentary PBAs found in Exp2 for $\beta = 0$ disappear in Exp5 and Exp6, cf. Supplementary Figure S11 (panels (e–l)); (2) in Exp6, during the long-term evolution after the TP and while subject to time-independent forcing, phase coherence is suddenly lost, cf. Supplementary Figure S11 (panels (i–l)); and (3) the TIID found in many cases in Exp2 is preserved under periodic perturbations, cf. Supplementary Figure S10 (panels (c, d)). These latter results are particularly unexpected in view of the less robust character of the other features observed in Exp1 and Exp2.

Finally, in Exp7 and Exp8, sensitivity to forcing period *T* was investigated. Here, the parameters take on the Exp1 values and the ramp steepness is held constant, with $\tau =800$ year; two amplitudes of the forcing perturbation were considered ($\beta = 0.05, 0.1)$, while the period varied over a wide range of values, $T = 1{-}100$ year. For very large periods, the tipping occurs at values close to those found in Exp1.

On the other hand, for periods *T* in the forcing that are comparable to the ROs’ typical time scale, a dramatic drop in the TP timing $t_{\mathrm{{tp}}}$ and corresponding forcing amplitude $G^{*}_{\mathrm{{tp}}}$ occurs, cf. Fig. 10. For two particular periods, the forcing amplitude at the tipping is well below the value required for the autonomous system to transition from the excitable to the self-sustained RO regime. Thus, we are in the presence of nonlinear-resonance-like behavior.

The strong possibility of rough parameter dependence raised by these results needs to be explored further by bringing to bear the tools needed to study the system’s associated transfer operator and its Ruelle–Pollicott resonances. More generally, the many interesting types of tipping effects obtained in this work should be further investigated.

Particularly intriguing is the dependence on model history, which seems to contradict the Markovian character of its governing equation (1). Memory effects have been studied with considerable attention in the climate sciences over the last decades (e.g.,^90–92^, and references therein). But in the present case, the memory seems to apply collectively to the single or multiple PBAs, and not so much to the individual trajectories. Some guidance for this situation may be available in the work of D. Mukhin and colleagues^93,94^, who studied regime transitions in an ENSO model with memory. A complementary way of studying regime transitions, in a paleoclimate context, can be found in^95^.

In conclusion, a wealth of interesting information was obtained in the present investigation on the TPs induced by parameter drift in an excitable system. We believe the results are of potential interest in several areas of climate dynamics. Earth system models of intermediate complexity or even more realistic climate models may experience tipping scenarios similar to those found herein; these scenarios could lend themselves to the kind of study exemplified in our work.

On the other hand, some scenarios found with the present simple model may not show up in more detailed models because of the many parameterizations needed to insure the numerical stability of the latter^16,96^. Our simple model could, if so, at least suggest the possible existence of such hidden scenarios and stimulate their investigation.

## Supplementary information

Supplementary Information.
