# Author Correction: Tipping points induced by parameter drift in an excitable ocean model

**DOI:** 10.1038/s41598-022-12470-4

**Published:** 2022-05-18

**Authors:** Stefano Pierini, Michael Ghil

**Affiliations:** 1 Department of Science and Technology, Parthenope University of Naples, Centro Direzionale, Isola C4,, 80143 Napoli, Italy; 2 CoNISMa, Rome, Italy; 3 Geosciences Department and Laboratoire de Météorologie Dynamique (CNRS and IPSL), École Normale Supérieure and PSL University, Paris, France; 4 Atmospheric and Oceanic Sciences Department, University of California at Los Angeles, Los Angeles, CA USA

Correction to: *Scientific Reports* https://doi.org/10.1038/s41598-021-90,138-1, published online 27 May 2021

The original version of this Article contained an error, as it did not mention the related work of Daruka and Ditlevsen (2016). As a result, Reference 70 was omitted and is listed below.

Daruka, I., & Ditlevsen, P. D. A conceptual model for glacial cycles and the middle Pleistocene transition. *Clim. Dyn.* **46**, 29–40, https://doi.org/10.1007/s00382-015-2564-7 (2016).

Reference 71 has also been added, and is listed below.

Riechers, K., Mitsui, T., Boers, N., & Ghil, M. Orbital insolation variations, intrinsic climate variability, and Quaternary glaciations. *Clim. Past.* **18**, 863–893, https://doi.org/10.5194/cp-18-863-2022 (2022).

As a result of the above changes, the References have been renumbered in the original Article and the Supplementary Information file.

In addition, in the Model and methods section, under the subheading 'The model and the experimental setup',

“Here *γ*, *α* and *β* are positive dimensionless constants, ω = 2*π/T*, while *R*_*τ*_*(t)* is the ramp function shown in Fig. 1, with *τ* = *t*_2_ − *t*_1_ and *t*_1_ = 200 year throughout the analysis; the explicit formula for *R*_*τ*_*(t)* is given in Supplementary Equation (S1).”

now reads:

“Here *γ,* *α* and *β* are positive dimensionless constants, *ω* = 2*π/T*, while *R*_*τ*_*(t)* is the ramp function shown in Fig. 1, with *τ* = *t*_2_ − *t*_1_ and *t*_1_ = 200 year throughout the analysis; the explicit formula for *R*_*τ*_*(t)* is given in Supplementary Equation (S1). A similar ramp—with a sigmoid, hyperbolic-tangent shape, rather than the trigonometric shape given by Equation (S1) herein—was used by Daruka and Dietlevsen^70^ in the study of the mid-Pleistocene transition (MPT) in the amplitude and mean period of the Quaternary era’s glacial–interglacial cycles. The MPT and the various approaches used to simulate and explain it are discussed in Section 4 and Appendix A of Riechers et al.^71^”

And in the Acknowledgements,

“Chris Jones kindly pointed out to us two recent papers relevant to R-tipping in excitable systems^32,33^. The authors wish to thank two anonymous reviewers, whose constructive comments helped improve the manuscript.”

now reads:

“Chris Jones kindly pointed out to us two recent papers relevant to R-tipping in excitable systems^32,33^. The authors wish to thank two anonymous reviewers, whose constructive comments helped improve the manuscript. István Daruka drew our attention to the similarity between the monotonic ramp function used in the MPT study^70^ and the one used in the present study.”

The original Article and the Supplementary Information file that accompanies the original Article, have been corrected.

