# Supplementary material for: Tipping points induced by parameter drift in an excitable ocean model
Source: Sci Rep. 2021 May 27;11:11126. doi: 10.1038/s41598-021-90138-1 (PMC8159979; doi:10.1038/s41598-021-90138-1)
Supplement: Supplementary file 1 — Supplementary Information. [file 41598_2021_90138_MOESM1_ESM.pdf]

Supplementary Information for: Tipping points induced by parameter drift in an excitable ocean model

Stefano Pierini[1,2*] **and Michael Ghil**[3,4]

[1]Department of Science and Technology, Parthenope University of Naples, Italy
[2]CoNISMa, Rome, Italy
[3]Geosciences Department and Laboratoire de Météorologie Dynamique (CNRS and IPSL), École Normale Supérieure and PSL University, Paris, France
[4]Atmospheric and Oceanic Sciences Department, University of California at Los Angeles, Los Angeles, California, USA
[*]stefano.pierini@uniparthenope.it

ABSTRACT

Numerous systems in the climate sciences and elsewhere are excitable, exhibiting coexistence of and transitions between a basic and an excited state. We examine the role of tipping between two such states in an excitable low-order ocean model. Ensemble simulations are used to obtain the model's pullback attractor (PBA) and its properties, as a function of a forcing parameter γ and of the steepness δ of a climatological drift in the forcing. The tipping time t_{tp} is defined as the time at which the transition to relaxation oscillations (ROs) arises: at constant forcing this occurs at $\gamma = \gamma_c$. As the steepness δ decreases, t_{tp} is delayed and the corresponding forcing amplitude decreases, while remaining always above γ_c. With periodic perturbations, that amplitude depends solely on δ over a significant range of parameters: this provides an example of rate-induced tipping in an excitable system. Nonlinear resonance occurs for periods comparable to the RO time scale. Coexisting PBAs and total independence from initial states are found for subsets of parameter space. In the broader context of climate dynamics, the parameter drift herein stands for the role of anthropogenic forcing.

Supplementary Figures

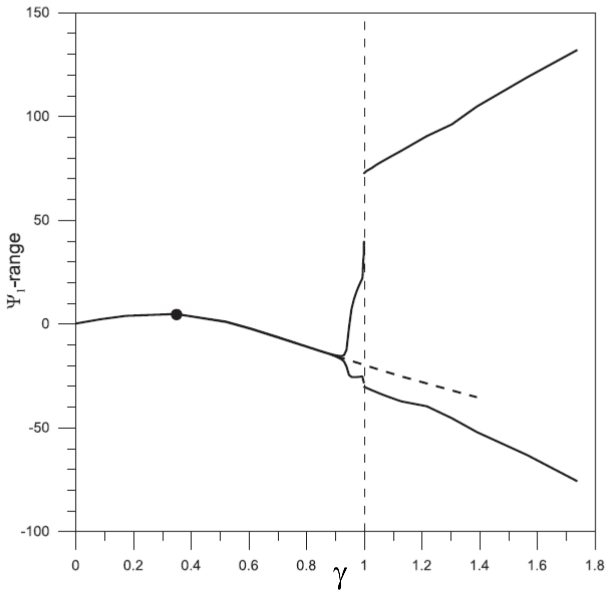

Supplementary Figure S 1. Bifurcation diagram of the autonomous system ($\alpha = \beta = 0$) showing the range of variability of Ψ_1 vs the normalized forcing amplitude γ; the filled circle indicates the Hopf bifurcation at $\gamma = 0.348$, while the black dashed line indicates the unstable steady state. The light dashed vertical line shows the sharp transition from small-amplitude oscillations to ROs that occurs at $\gamma = \gamma_c = 1$. Adapted from [62] (© American Meteorological Society; used with permission).

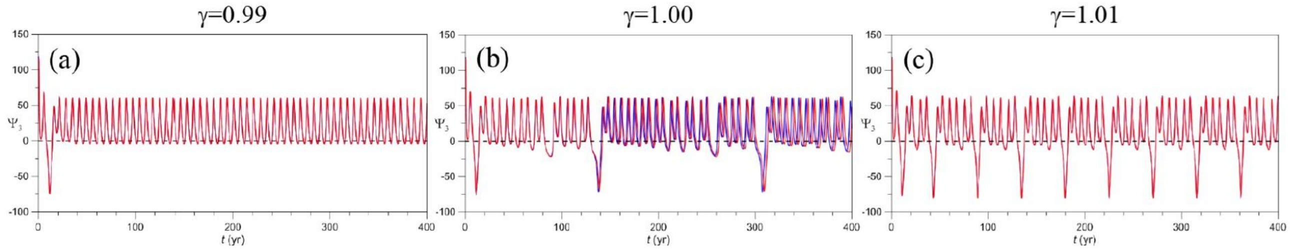

Supplementary Figure S 2. Critical transition in the autonomous system at $\gamma = \gamma_c = 1$. Evolution of $\Psi_3(t)$ for two trajectories (blue and red lines) started at nearby points for (a) $\gamma = 0.99$, (b) $\gamma = 1$, and (c) $\gamma = 1.01$. Adapted from [66].

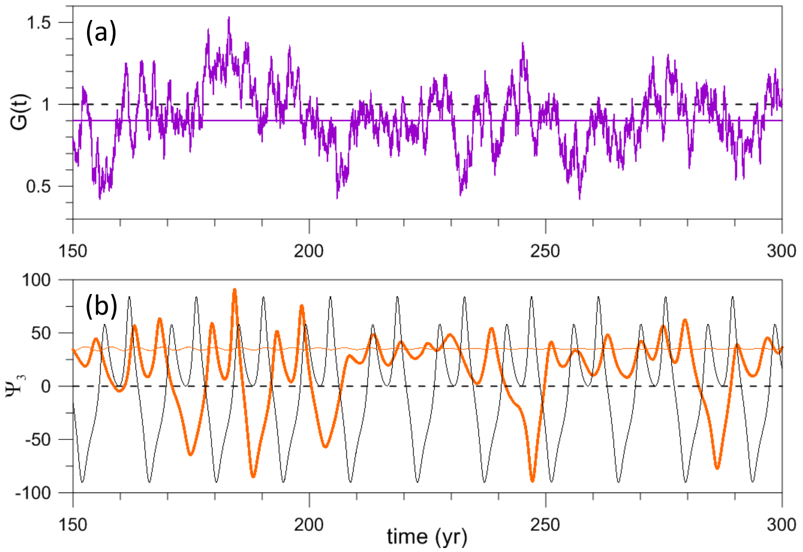

Supplementary Figure S 3. Relaxation oscillation (RO) excitation by noise. (a) Time dependence of the factor $G(t)$ in the external forcing with $\gamma = 0.9$ (solid purple line) plus a red noise $\varepsilon\zeta(t)$ with $\varepsilon = 0.2$ and $T_d = 2$ yr. (b) Heavy orange line: model response in terms of Ψ_3. The light orange and black lines show the autonomous response for $\gamma = 0.9$ and $\gamma = 1.2$, respectively, as already reported in Figs. 2(b,d).

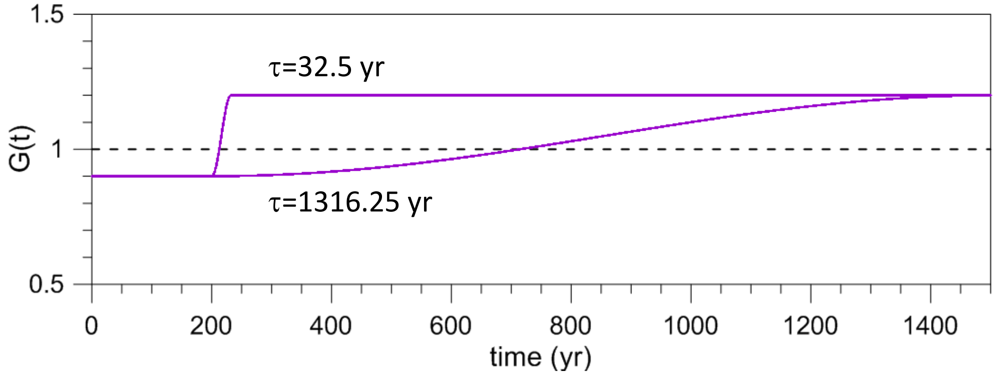

Supplementary Figure S 4. Time dependence of the factor $G(t)$ in the forcing for the smallest and largest τ-value used in Exp1.

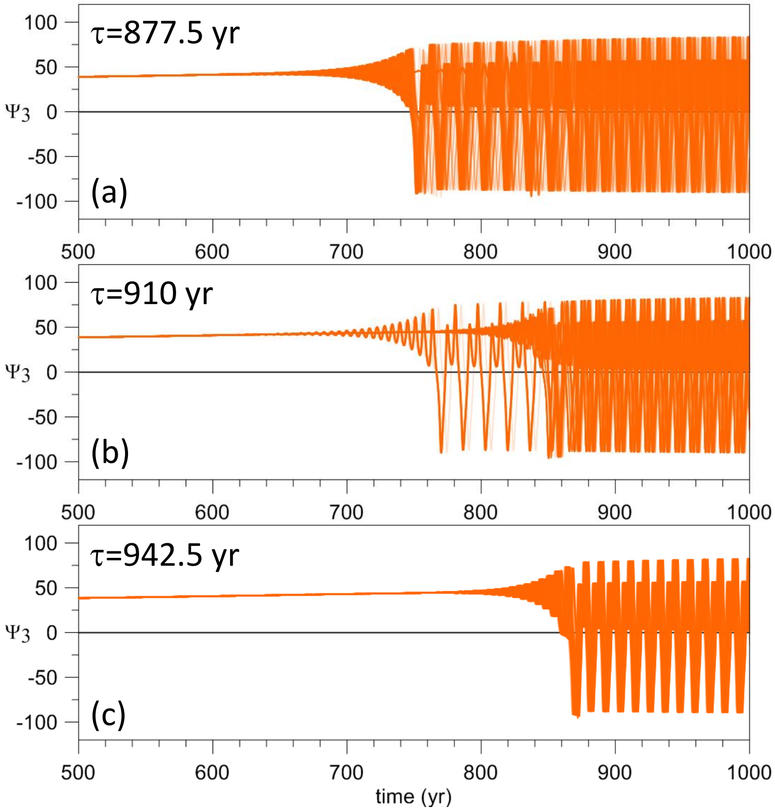

Supplementary Figure S 5. Nonmonotonic TP-dependence on ramp duration τ. This dependence is illustrated by the $\Psi_3(t)$ time series for the ESs of Exp1, with three distinct τ-values: (a) $\tau = 877.5$ yr; (b) $\tau = 910$ yr; and (c) $\tau = 942.5$ yr. These three cases correspond to the filled circles for the points $P_1 - P_3$ in Fig. 5(b).

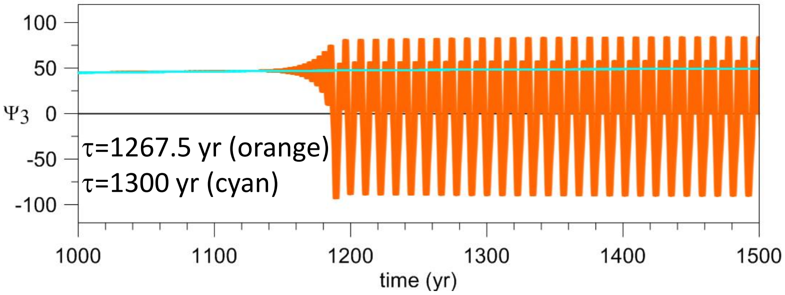

Supplementary Figure S 6. Same as Figs. S5(a–c), but for $\tau = 1\,267.5$ yr (orange lines) and $\tau = 1\,300$ yr (cyan lines). The orange ES corresponds to the filled circle P_4 in Fig. 5(b); the cyan ES is unstable, as shown in Fig. S7.

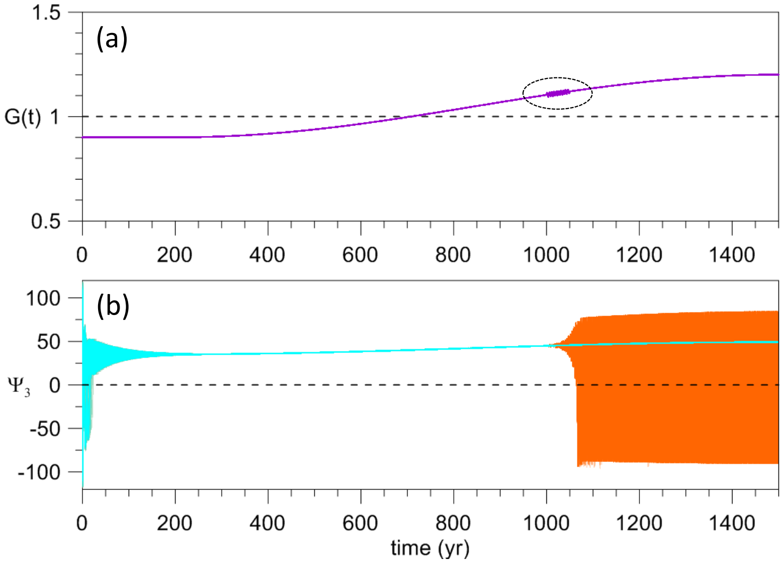

Supplementary Figure S 7. Nonlinear instability of an ES with the parameter values of Exp1, cf. Table 1; this ES corresponds to $\tau = 1\ 300$ yr but with a very small periodic perturbation superimposed on the forcing $G(t)$. (a) Evolution of $G(t)$, with $\beta = 0.01$ and $T = 5$ yr, acting on the interval $1\ 000 \leq t \leq 1\ 050$ yr; the periodic perturbation is highlighted by the oval. (b) Evolution of the model's $\Psi_3(t)$ component: orange lines — for the $G(t)$ in panel (a); cyan lines: for the same $G(t)$ but with $\beta \equiv 0$. The latter ES belongs to Exp1 and it corresponds to the filled circle P_4 of Fig. 5(b); it is also shown in Fig. S6 by cyan lines.

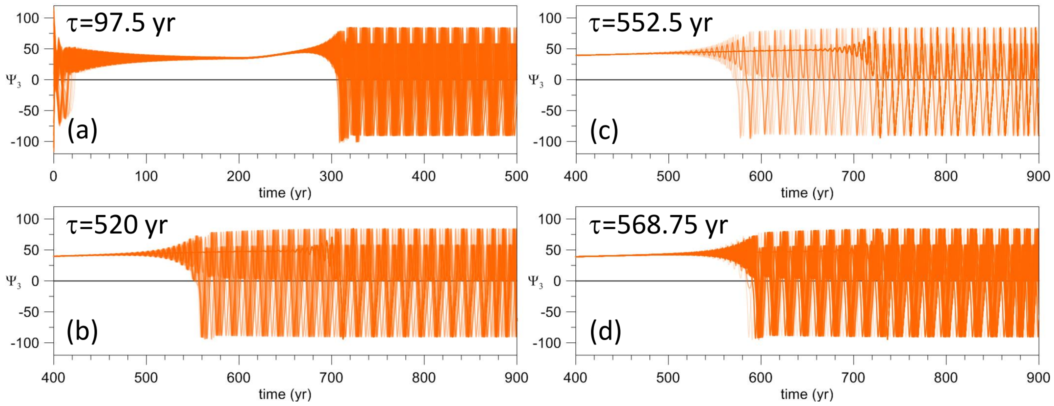

Supplementary Figure S 8. Same as Figs. S5 and S6 but for the ESs of Exp1 corresponding to the red bars in Fig. 7: (a) $\tau = 97.5$ yr; (b) $\tau = 520$ yr; (c) $\tau = 552.5$ yr; and (d) $\tau = 568.75$ yr.

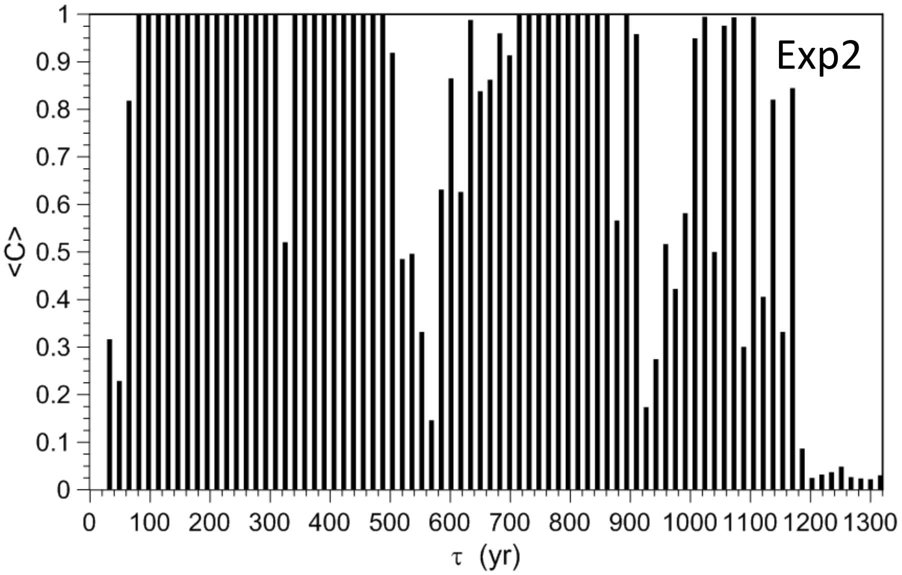

Supplementary Figure S 9. Same as Fig. 7 but for Exp2.

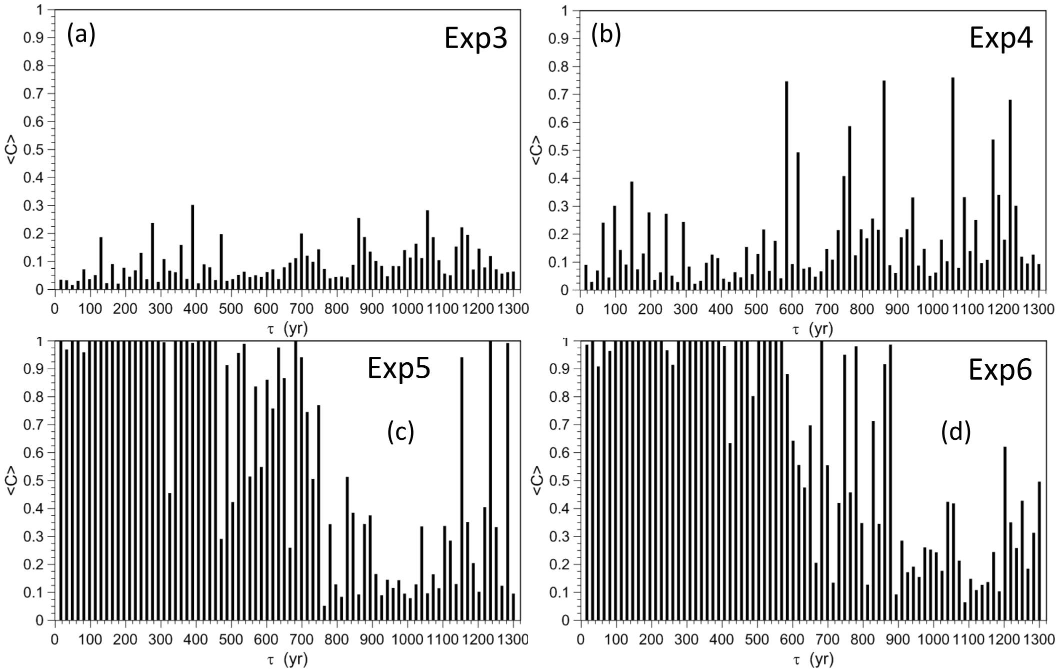

Supplementary Figure S 10. Same as Fig. 7 but for Exp3–Exp6.

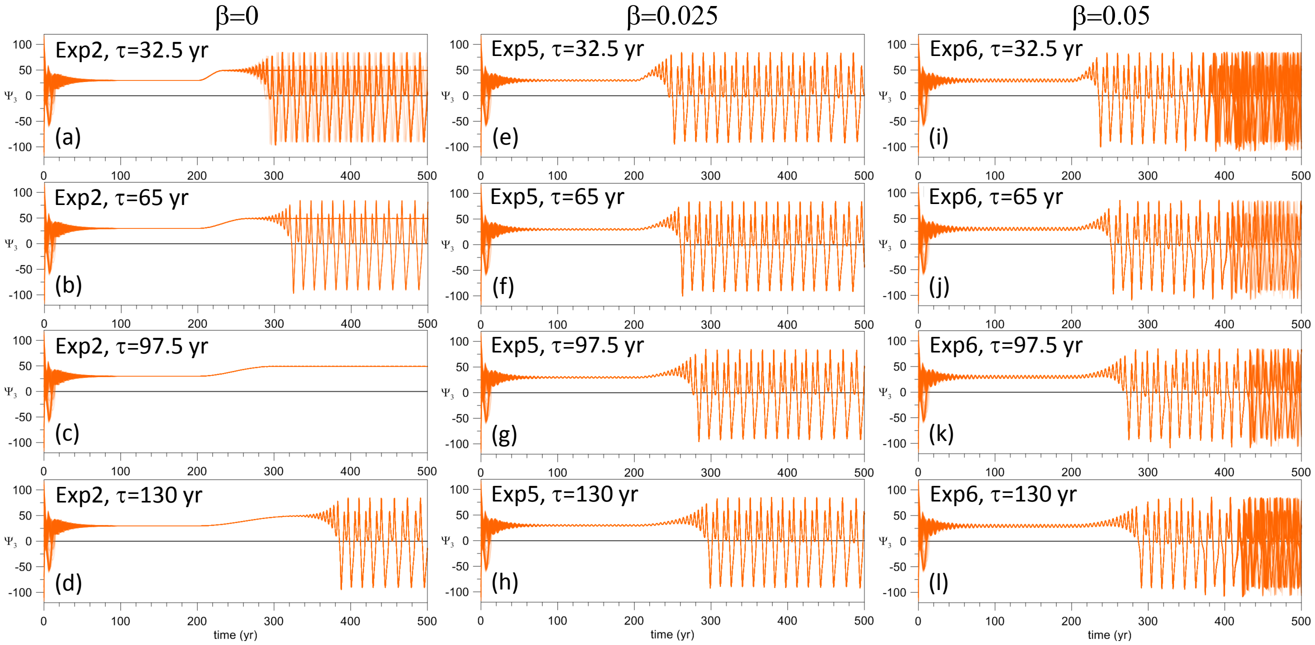

Supplementary Figure S 11. ESs from Exp2 ($\beta = 0$, left column), Exp5 ($\beta = 0.025$, central column) and Exp6 ($\beta = 0.05$, right column), represented by time series of $\Psi_3(t)$ of the corresponding ESs. The successive panels in each column correspond to $\tau = 32.5,\ 65,\ 97.5$ and 130 yr.

Supplementary discussion

Supplementary Information on: "The model and the experimental setup"

The ramp function $R_\tau(t)$ present in Eq. (2) and shown in Fig. 1 is defined as:

$$t < t_1 : R = 0; \quad t_1 < t < t_2 : R = \frac{1}{2}\left\{1 + \cos\left[\pi\left(\frac{t_1 - t}{\tau} + 1\right)\right]\right\}; \quad t_2 < t : R = 1. \tag{S1}$$

The behavior of the model's autonomous version, with $\alpha = \beta = 0$ in Eq. (2), is described by the bifurcation diagram of Fig. S1: it shows the minimum and maximum values attained by Ψ_1 after spinup vs. the control parameter γ. The diagram was obtained in [62] by using a "poor man's continuation" method ([7] and references therein), i.e., by performing many forward time integrations with different values of γ, all initialized from rest, to attain the corresponding asymptotic solutions plotted in the figure herein.

For small γ, i.e., low wind stress, the steady-state, double-gyre solution is stable. A supercritical Hopf bifurcation to a stable limit cycle occurs at $\gamma = 0.348$ but the amplitude of the latter is rather small up to $\gamma \cong 0.9$, after which it increases quite rapidly; the filled circle in the figure indicates the original Hopf bifurcation. The critical value $\gamma = \gamma_c = 1$ corresponds to a TP that marks the abrupt transition from small-amplitude limit cycles to large-amplitude self-sustained ROs. Besides these limit cycles, ESs show the existence of stable fixed points emanating from very small basins of attraction. Moreover, Pierini et al. [66] showed that the system enters the chaotic regime at $\gamma = \gamma_0 = 1.3475$.

Figure S2 [66] clarifies the character of the critical transition at $\gamma = 1$. For $\gamma = 0.99$, shown in Fig. S2(a), the $\Psi_3(t)$ time series of two trajectories (red and blue lines) that start out near each other exhibit small-amplitude oscillations in both, with no visible divergence. For $\gamma = 1.01$ in panel (c), the two trajectories still coincide, although larger amplitude ROs appear at regular intervals in the periodic signal. Note that, for $\gamma = 1$ in panel (b), the two trajectories differ, yielding small- and large-amplitude oscillations that alternate at irregular intervals; in fact, it was shown [66] that chaotic dynamics occurs in an extremely restricted range that separates the two different types of limit cycles. This critical transition may be due to a canard explosion [72].

Figure S3 provides an example of ROs that are excited by noise when the system is in the excitable regime $\overline{\gamma_s} \leq \gamma \leq \gamma_c$, where $\overline{\gamma_s}$ denotes the expectation of the random variable γ_s. The forcing is the same as in Fig. 2(a), with $\gamma = 0.9$ (straight purple line), but now a normalized Ornstein-Uhlenbeck noise $\varepsilon\zeta(t)$ is added to $G(t)$ of Eq. (2), in which $\varepsilon = 0.2$ and ζ has a decorrelation time $T_d = 2$ yr. A realization of $G(t)$ in the time interval 150–300 yr is shown in Fig. S3(a) (squiggly purple line), while in Fig. S3(b) the system's response is shown by the heavy orange line.

For the sake of comparison, the light orange and black lines show the autonomous response for $\gamma = 0.9$ and $\gamma = 1.2$, respectively, as already reported in Figs. 2(b,d). Thus, the heavy orange line will tend to the light one as the noise vanishes, $\varepsilon \to 0$. The excited ROs of Fig. S3(b) are quite similar to the self-sustained ROs of the autonomous case, despite the large difference in the climatological value of the forcing, i.e. $\gamma = 0.9$ vs. 1.2. The only difference is in the greater intermittency of the large minima in $\Psi_3(t)$ in the presence of the noise, but the latter has little effect on the local smoothness of the solution [39]. Naturally, the two-dimensional evolutions of the streamfunction fields $\psi(\mathbf{x},t)$ of the excited and self-sustained ROs are very similar as well; the latter are not shown here, but see [62] and Fig. 7 therein.

Finally, for the sake of clarity Fig. S4 shows, as an example, the time dependence of the forcing $G(t)$ for the smallest and largest τ-value used in Exp1.

Supplementary Information on: "Results: the basic numerical experiment"

In Figs. S5-S8 we investigate in further detail the anomalous behavior noted in Fig. 5. Figure S5 shows the 80 time series of $\Psi_3(t)$ for the three ESs that correspond to the points $P_1 - P_3$ of Fig. 5(b).

At P_1, $\tau = 877.5$ yr and virtually all the ensemble members yield the first RO at $t_{tp} \cong 750$ yr (Fig. S5a). At P_2, $\tau = 910$ yr and t_{tp} changes slightly to $t_{tp} \cong 770$ yr, but only a few ensemble members exhibit their first RO at that TP, cf. Fig. S5(b): for all the remaining members, the first RO appears much later, at $t \cong 850$ yr. It is this group of members that determines the subsequent evolution of t_{tp}: at P_3, with $\tau = 942.5$ yr, all the members yield, in fact, the first RO at $t_{tp} \cong 870$ yr, cf. Fig. S5(c). After P_3, the behavior is the same as the one before P_1, until a further jump at $\tau \cong 1\,140$ yr occurs due to a similar phenomenon.

It follows that this anomalous dependence of t_{tp} and G_{tp} upon τ is associated with the sudden appearance of two clusters in the ES and the equally sudden disappearance of the first cluster, which leads to an abrupt forward shift of the TP, as defined in this study. The coexistence of such clusters suggests the possibility of two distinct local PBAs, as discussed in [66]. The authors showed there that the dissipative model of Eq. (1) possesses a unique global PBA but suggested that the numerically found coexistence of two local PBAs with distinct stability properties was consistent with the well-studied simpler case of a Van der Pol–Duffing oscillator.

Another interesting aspect that is worth considering is the sudden lack of TPs for $\tau \sim 1\,300$ yr. In Fig. 5(b), P_4 denotes the ES (orange lines of Fig. S6) with the highest τ-value, $\tau = 1\,267.5$ yr, that still yields ROs. For greater values, all trajectories collapse onto an extremely small-amplitude limit cycle, as illustrated by the cyan lines of Fig. S6, which corresponds to $\tau = 1\,300$ yr. This filamentary PBA is, however, nonlinearly unstable, as seen in the perturbed ES shown in Fig. S7. Here, a very small-amplitude periodic perturbation acting over a short time interval on the forcing $G(t)$, cf. Fig. S7(a), makes the model transition from the cyan to the orange trajectories in Fig. S7(b).

The cyan and orange ensembles of trajectories in Fig. S7(b) appear therewith as estimates of two coexisting PBAs with very different stability properties. Additional numerical experiments (not shown) confirm that not even much stronger perturbations cause the system to shift from the RO-PBA (orange) to the small-size, sinusoidal PBA (cyan).

The four red bars of Fig. 7 refer to the ESs reported in Fig. S8. For $\tau = 97.5$ yr, the very small value of $\bar{C} \simeq 0.01$ indicates a notable phase spread of the ROs (Fig. S8(a)), while the higher value for $\tau = 520$ yr indicates a more limited spread (Fig. S8(b)). For $\tau = 552.5$ yr, the value $\bar{C} = 0.86$ indicates an appreciable clustering (Fig. S8(c)), which disappears for a small increment of τ, up to $\tau = 568.75$ yr, to yield a very small $\bar{C} = 0.063$ in Fig. S8(d).

Finally, Fig. S9 shows the dependence of the number $\bar{C}$ of trajectory clusters on τ for Exp2 (see the main text for a discussion).

Supplementary Information on: "Sensitivity to periodic perturbations (Exp3–Exp6)"

Figure S10 provides interesting results concerning the dependence of the phase distribution of ROs on the presence and amplitude of periodic forcing. Figure S10(a) for Exp3 displays a more uniform distribution of $\bar{C}$-values than in Fig. 7 for Exp1, with the high values being reduced and the very small ones being increased. Note that the disappearance of values close to unity at $\tau \simeq 1\,300$ yr is obvious in view of the instability of the corresponding filamentary PBAs, as seen in Fig. S7. However, for a higher perturbation amplitude, several cases with a fairly high value of $\bar{C}$ appear, as found in Fig. S10(b) for Exp4. Hence, although t_{tp} and G^*_{tp} are almost equal in Exp3 and Exp4, cf. Fig. 5, the RO phases in Exp4 are more prone to cluster into STs than in Exp3.

Another intriguing result emerges when comparing $\bar{C}$ of Exp5 and Exp6 in Figs. S10(c,d) with that of Exp2 in Fig. S9. The TIID noticed in Exp2 is generally preserved in the presence of periodically perturbed forcing up to $\tau \simeq 800$ yr; on the other hand, $\bar{C}$ undergoes a notable reduction for higher values of τ. Again, the perturbation amplitude does not appear to play a major role inasfar as this effect is concerned: compare panels (c) and (d) of Fig. S10. We thus conclude that TIID is a robust feature for high-to-moderate forcing drift rates δ but tends to disappear for lower α (Exp3 and Exp4, with $\alpha = 0.3$), and hence δ.

Finally, we complement the analysis with the study of four particularly interesting ESs for each one of Exp2, Exp5 and Exp6; these ESs are carried out in steps of $\Delta\tau = 32.5$ yr and plotted in Fig. S11 in three columns of four panels each. For Exp2, with no periodic forcing, in the first column of Fig. S11, we notice four interesting features:

- For panels (a,b,d), the TP is reached after the end of the ramp, as can also be seen in Fig. 5(a), since the red line that corresponds to Exp2 there lies in the gray area. A TP being reached after the forcing strength has increased and achieved its maximum level appears to be novel, and it deserves further investigation elsewhere.
- In panel (b), two single trajectories emerge (see the corresponding definition after Eq. (5)). This represents the coexistence of two disjoint PBAs with distinct properties: nearly constant vs periodic.
- For $\tau = 97.5, 130$ yr in panels (c) and (d), TIID occurs; this is also revealed by the corresponding values $\bar{C} = 1$ in Fig. S9.
- For $\tau = 97.5$ yr in panel (c), in particular, the filamentary nature of the PBA implies the absence of a TP; this is also revealed by the absence of a filled circle corresponding to that value of τ on the red line of Fig. 5(a).

The features listed above, though, are not robust, as shown in the second column of Fig. S11. The only striking feature that survives a perturbation by the small periodic component present in Exp5 is the occurrence of TIID, as confirmed by the values of $\bar{C} \cong 1$ for the corresponding τ-values in Fig. S10(c).

Finally, if the periodic perturbation's amplitude doubles, as shown for Exp6 in the third column of Fig. S11, no substantial changes in the TP timing t_{tp} occur, but a novel feature does emerge: namely, the ROs undergo irregular oscillations, while still maintaining TIID.

Moreover, 160–180 yr after t_{tp}, phase coherence is rapidly lost. Remarkably, this coherence loss occurs while the forcing is constant in time, having passed the ramp in $R_\tau(t)$ already; once again, this rather unexpected feature deserves further analysis in future work.
